# Pilot Study to Quantify Palladium Impurities in Lead-like Compounds Following Commonly Used Purification Techniques

**DOI:** 10.1021/acsmedchemlett.1c00638

**Published:** 2022-01-20

**Authors:** Maria Chatzopoulou, Katrina S. Madden, Liam J. Bromhead, Christopher Greaves, Thomas J. Cogswell, Solange Da Silva Pinto, Sébastien R. G. Galan, Irene Georgiou, Matthew S. Kennedy, Alice Kennett, Geraint Apps, Angela J. Russell, Graham M. Wynne

**Affiliations:** † Department of Chemistry, Chemistry Research Laboratory, University of Oxford, Oxford OX1 3TA, United Kingdom; ‡ Department of Pharmacology, University of Oxford, Mansfield Road, Oxford OX1 3PQ, United Kingdom; § CEMAS, Imperial House, Oaklands Business Centre, Oaklands Park, Wokingham, Berkshire RG41 2FD, United Kingdom; ∥ OxStem Limited, Midland House, West Way, Botley, Oxford OX2 0PH, United Kingdom

**Keywords:** Palladium, trace impurities, lead optimization, screening, assay interference, purification, metal scavenging

## Abstract

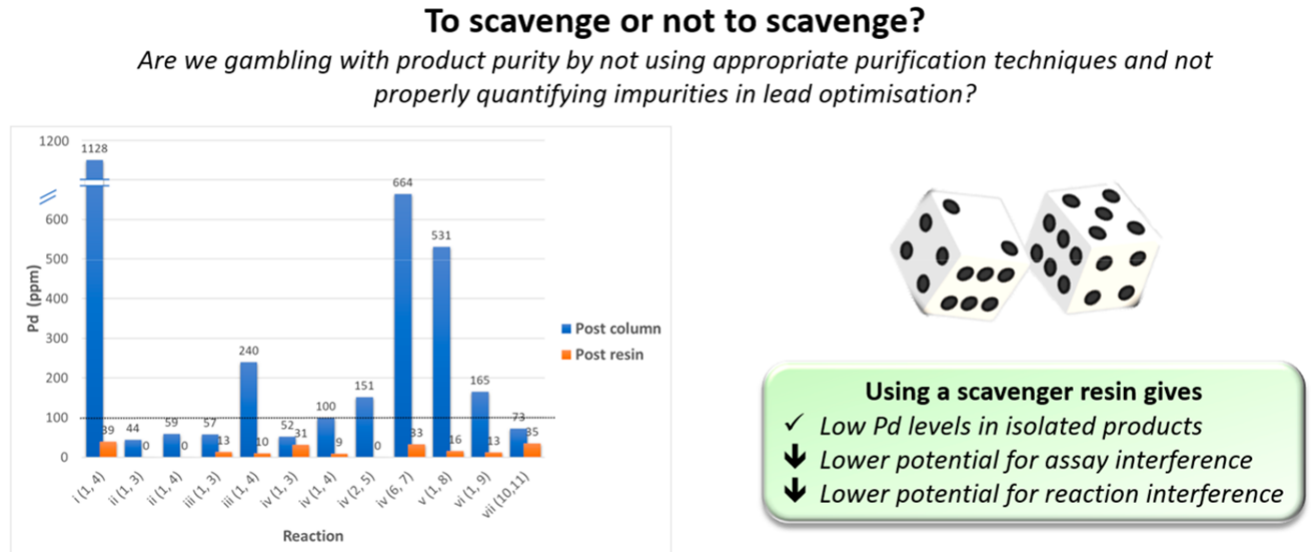

Palladium-catalyzed reactions are among the most commonly used procedures in organic synthesis. The products have a range of uses, including as intermediates in total synthesis and as screening compounds for drug discovery or agrochemical projects. Despite the known and potentially deleterious effects of low-level metal impurities in biological assays, the quantification of metal remaining in reaction products to verify the effective removal of the transition element is rarely reported. Using palladium as an exemplar, we describe a pilot study that for the first time quantifies residual metal levels in reaction products following increasingly rigorous purification protocols. Our results demonstrate that significant levels of residual palladium can remain in isolated reaction products following chromatographic purification, and only by using a subsequent metal scavenging step are they reliably reduced to a low level. Finally, we provide a set of simple guidelines that should minimize the potential for issues associated with residual palladium in reaction products.

Assessing the identity, yield, and purity of newly prepared compounds is a routine and fundamentally important component of organic synthesis.^1^ However, problems can arise relating to either the authenticity of the target compound itself or the influence of the experimental/analytical conditions.^2,3^ Several recent publications have described issues of this type and their impact.^4−9^

During drug development, high purity and low batch-to-batch variability in compound samples is essential. Detailed quality criteria regarding API (active pharmaceutical ingredient) purity and trace-metal levels have been published by regulators to aid developers as they bring new drugs to market.^10,11^ Trace contaminants differ from chemical impurities directly connected to the synthesis of the API (e.g., products from side reactions) and can arise from residual catalysts or reagents used during synthesis or as an impurity leached from equipment during manufacturing.^12,13^

During lead optimization, quality assessment is vital to verify that the biological effect seen in an assay is due to the compound itself^14^ and not an impurity^15,16^ and so that any undesired effects seen (e.g., selectivity, toxicology, safety) can be unambiguously attributed to the test article. Whereas it is usually straightforward to quantify impurities that are present at higher levels (1–5 mol %) using conventional techniques such as nuclear magnetic resonance (NMR), trace-level impurities (<1 mol %) such as metal residues arising from catalysts can be “silent” because they are present at levels below instrument limits of detection.

The quantification and proven removal of low-level/trace impurities resulting from the use of metals in synthesis have received little attention in the mainstream medicinal chemistry literature.^17^ This is surprising given the frequent use of transition element catalysis,^18−22^ along with recent notable examples where the presence of trace impurities has proven problematic.^23−26^ The deleterious effects of trace-metal impurities on high-throughput screening (HTS) readouts are also well described.^27−31^ The fact that assay interference can occur during lead optimization seems unappreciated,^15,32^ and aside from process development chemists,^33−39^ few chemists publish proof that their reaction products are metal-free (Figure S1 and Tables S1 and S2).^40^

Numerous metal-scavenging protocols and reagents exist,^41,42^ and quantitative analysis is possible for a wide range of trace elements. ICP-MS (inductively coupled plasma mass spectrometry) is often used where sufficient material (10–20 mg) is available,^43^ whereas techniques such as fluorescence detection or X-ray fluorescence (XRF) may be preferable where the use of smaller sample quantities is desirable.^44−46^ More recently, a simpler methodology has been published using a fluorometric readout and equipment that should be readily available in the majority of laboratories.^47^

To address this paucity of data, we have undertaken a pilot study using palladium because it is the transition metal that is among the most commonly utilized in contemporary medicinal and process chemistry.^22,48,49^ We have quantified residual palladium levels in reaction products using ICP-MS in various different stages of the workup/purification process using increasingly rigorous methods.

We have focused on a small range of well-used reactions and reagents involving palladium-based catalysts.^50^ The indole scaffold is frequently described in contemporary medicinal chemistry projects,^51−54^ so 5-bromo-indoles **1** and **2** (Scheme 1) were used as the halogenated coupling partners. Suzuki–Miyaura and Buchwald–Hartwig cross-couplings along with palladium-on-carbon reduction were selected for study. Coupling partners were used that resulted in reaction products containing functionalities commonly seen in medicinal chemistry projects. Boronic acid/ester species giving rise to a 4-fluorophenyl containing **3** and *N*-methyl pyrazoles **4** and **5** for the Suzuki–Miyaura coupling were employed in addition to morpholine **8** and 4-fluoroaniline **9** derivatives for the Buchwald–Hartwig coupling. This scaffold was also used for the palladium-on-carbon reduction, employing 5-nitroindole **10**.

**Scheme 1:**
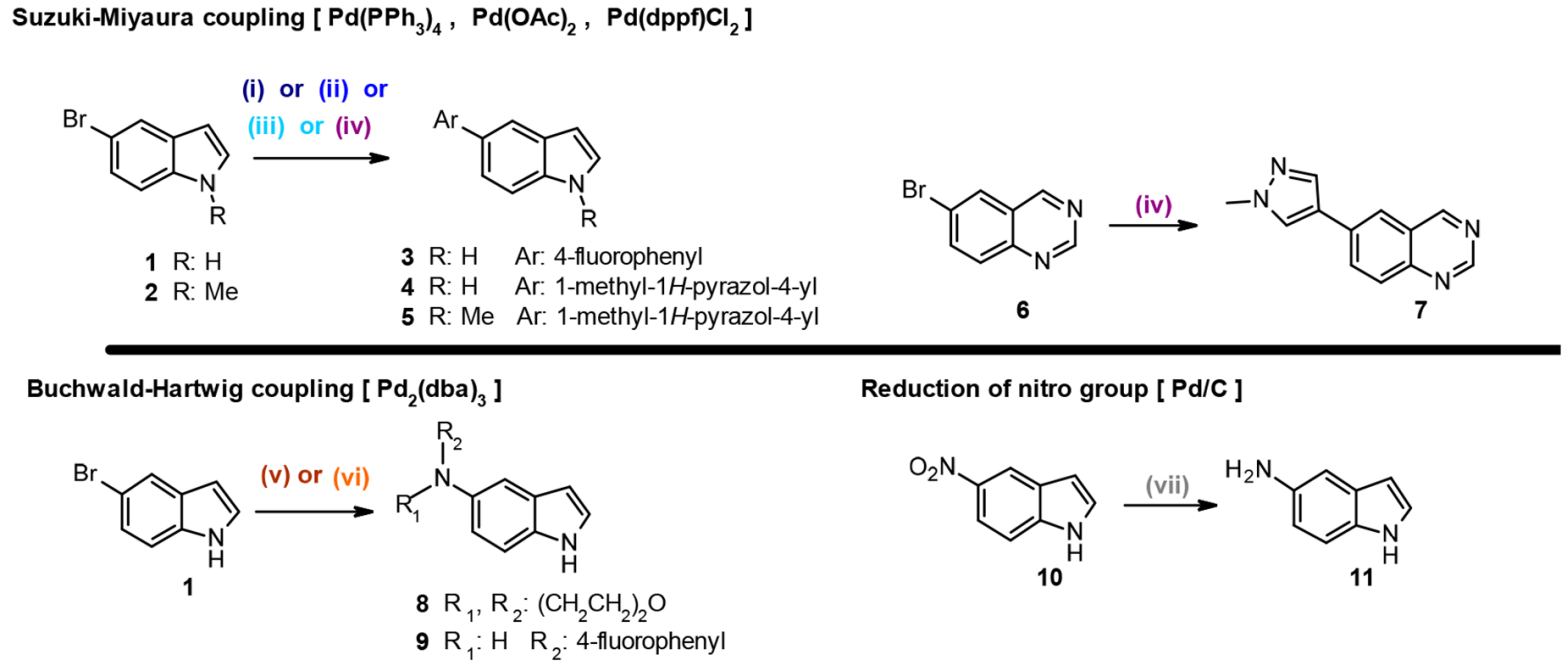
Reagents and conditions. (i) **1**, boronate species, Pd(PPh_3_)_4_, K_2_CO_3_, DME/H_2_O, 80 °C, 5 h; (ii) **1**, boronate species, Pd(OAc)_2_, PPh_3_, K_2_CO_3_, DME/H_2_O, 80 °C, 5 h; (iii) **1**, boronate species, Pd(OAc)_2_, SPhos, K_2_CO_3_, DME/H_2_O, 80 °C, 5 h; (iv) **2** or **6**, boronate species, Pd(dppf)Cl_2_, K_2_CO_3_, DME/H_2_O, 80 °C, 5 h; (v) morpholine, Pd_2_(dba)_3_, RuPhos, LHMDS, THF, 65 °C, 24 h; (vi) 4-fluoroaniline, Pd_2_(dba)_3_, BrettPhos, LHMDS, THF, 65 °C, 24 h; (vii) H_2_, 10% Pd/C, EtOH, RT, 24 h.

The building blocks utilized were selected so that the products would not cause unusual challenges in purification or be poorly soluble. The compound complexity and a higher number of heteroatoms could potentially affect the palladium retention.^55^ Therefore, along with indoles **1** and **2**, an alternative heterocyclic scaffold was included that has been well utilized in medicinal chemistry, bromoquinazoline **6**.^52,56^

Different palladium sources were used for each reaction type, with multiple catalyst/ligand combinations (Scheme 1 and Table 1). For the Suzuki–Miyaura reaction,^57^ four different sets of conditions (i–iv) were used,^58−61^ for the Buchwald–Hartwig,^62^ two variants (v and vi) were selected,^63^ and for heterogeneous catalytic reduction, single protocol (vii) was employed.

**Table 1:** Palladium Sources and Additives Used

reaction code	palladium source	additive
i	Pd(PPh_3_)_4_	
ii	Pd(OAc)_2_	PPh_3_
iii	Pd(OAc)_2_	SPhos
iv	Pd(dppf)Cl_2_	
v	Pd_2_(dba)_3_	RuPhos
vi	Pd_2_(dba)_3_	BrettPhos
vii	10% Pd/C	

In considering a target level of palladium impurity to set as an initial “maximum” for lead-like compounds, there are no published comparators, so we were guided by the FDA specifications for clinical-grade APIs, which is set at 100 μg/day orally.^11,64^ For compounds synthesized during lead optimization, which are at a much earlier stage of development, a level of 100 ppm (significantly higher than that required for a clinical stage API) appeared to represent an acceptable compromise between rigor and practicality. This is a level that should be achievable in all chemical laboratories and require no special equipment or reagents.

In the three different reaction classes evaluated, the residual palladium in reaction products varied from low levels (<100 ppm) to extremely high levels (>5000 ppm) depending on the palladium source and the extent of purification that had been employed (Figure 1A–C; also Tables S3A and S4A–C).

**Figure 1:**
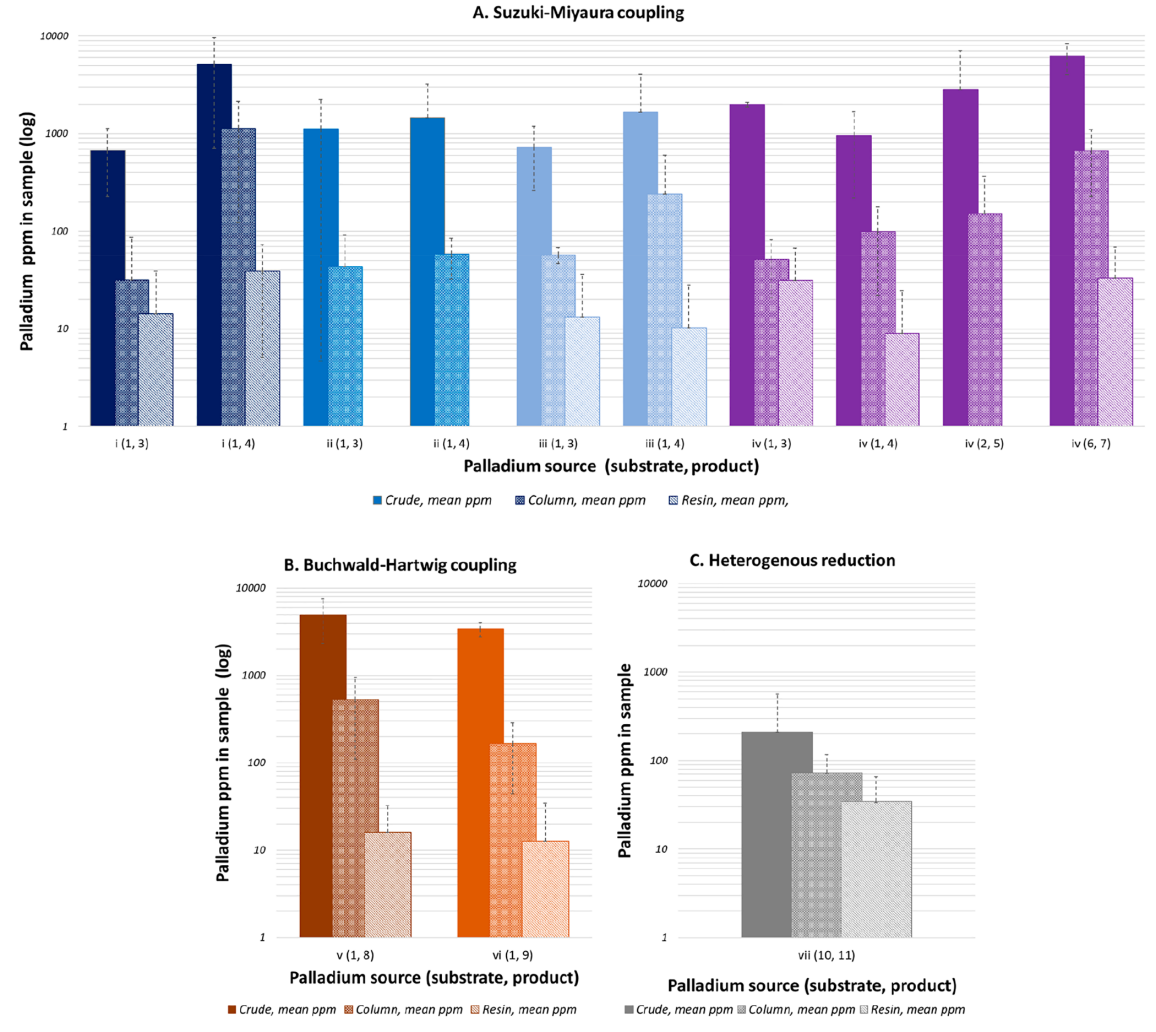
Residual palladium levels in reaction samples following (A) the Suzuki–Miyaura reaction, (B) the Buchwald–Hartwig reaction, and (C) metal-on-carbon reduction (reaction *n* = 3; SD = dashed lines; log scale).

Where purification consisted of only an aqueous workup, the palladium levels varied considerably (Figure 2) and were typically high (>1000 ppm, Table S5A).

**Figure 2:**
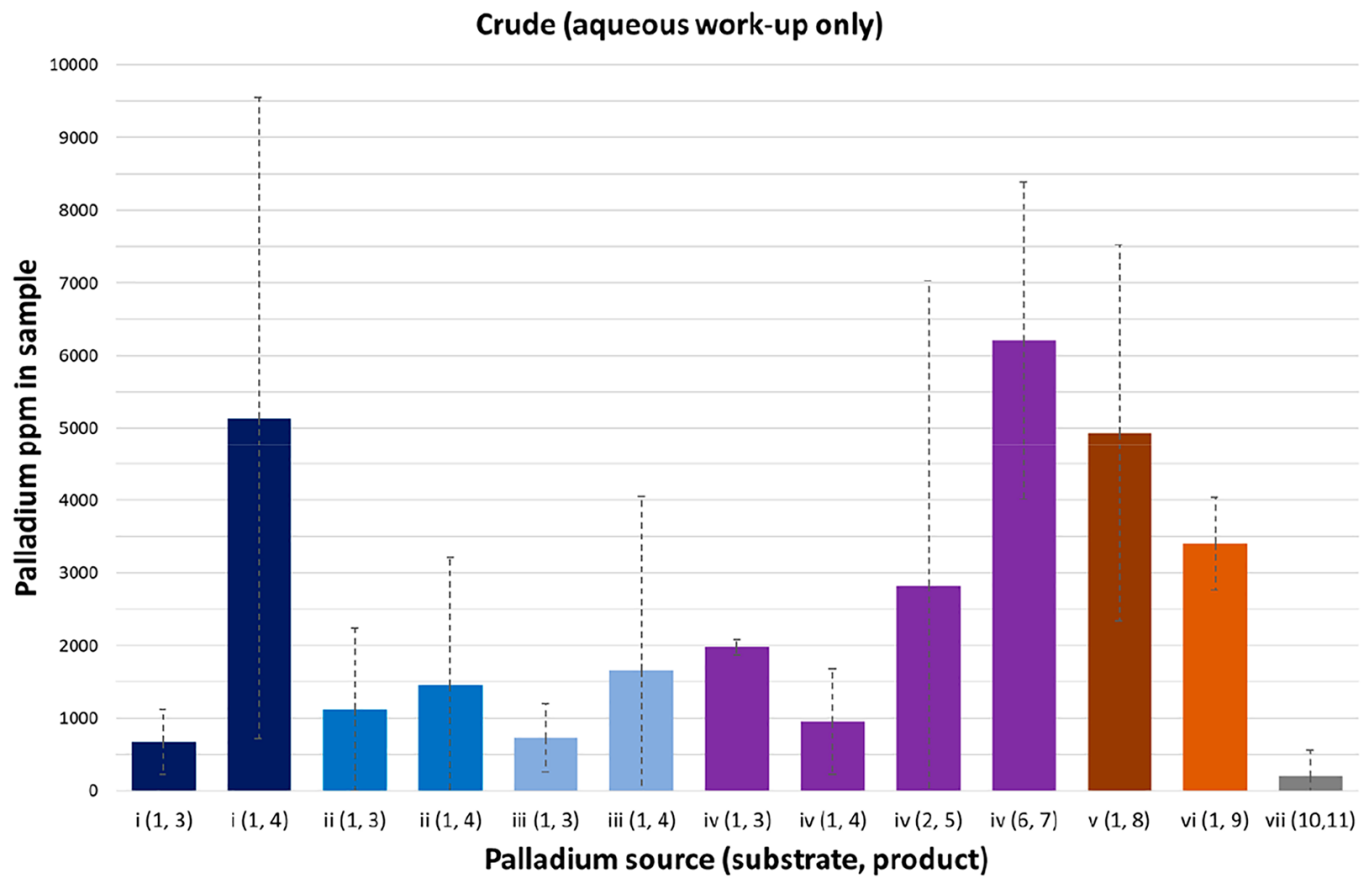
Residual palladium levels in crude reaction samples, aqueous workup only (reaction *n* = 3; SD = dashed lines).

Residue levels ranged from below the limit of quantification, for example, the nitro reduction reaction, palladium source (vii), to ∼5000 ppm for other palladium sources, for example, Suzuki–Miyaura i(1,4) and iv(6,7) and Buchwald–Hartwig v(1,8). Within different reaction runs, large standard deviation (SD) values were noted for palladium sources i(1,4) and iv(2,5) despite the use of the same batches of reagents and a standardized experimental procedure. Overall, when different reaction conditions were used for the same transformation, all Suzuki–Miyaura reactions (palladium sources (i)–(iv)), or Buchwald–Hartwig couplings (palladium sources (v), (vi)), varying levels of residual palladium and high SD values were seen that did not correlate with any specific experimental variable.

Palladium levels in samples purified by flash column chromatography also covered a significant range (Figure 3).

**Figure 3:**
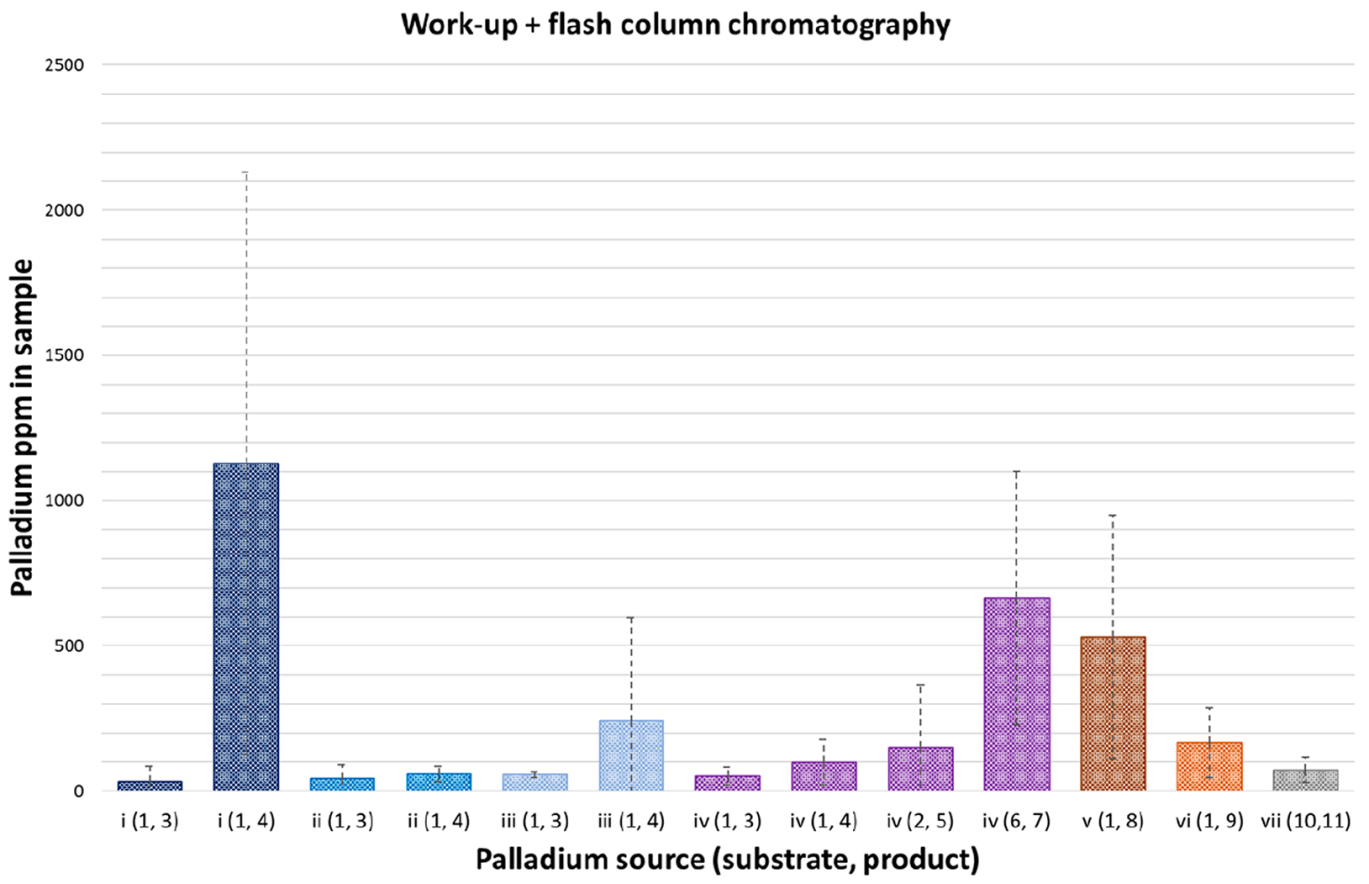
Residual palladium levels in reaction samples following automated (Biotage) flash chromatography (reaction *n* = 3; SD = dashed lines).

The highest levels were measured from Suzuki–Miyaura reaction i(1,4) at over 1000 ppm in 2/3 runs. The raw data show two of the experimental replicates (conducted by different chemists) as being >1000 ppm, suggesting that this was reagent-related rather than being procedural or operator-based (Table S5B). Column chromatography was effective in removing the majority of the residual palladium (to <100 ppm when averaged over the individual runs) in over half of the reaction products studied; however, >100 ppm levels were found in 14/39 of the individual samples. On average, comparing crude and post-column samples, ∼90% of the residual palladium was removed from crude samples using flash chromatography (Table S3B).

Palladium levels in products that had been successively purified using column chromatography and Si-TMT (2,4,6-trimercaptotriazine silica gel) scavenging resin all had low levels (<100 ppm) of residual palladium (Figure 4).

**Figure 4:**
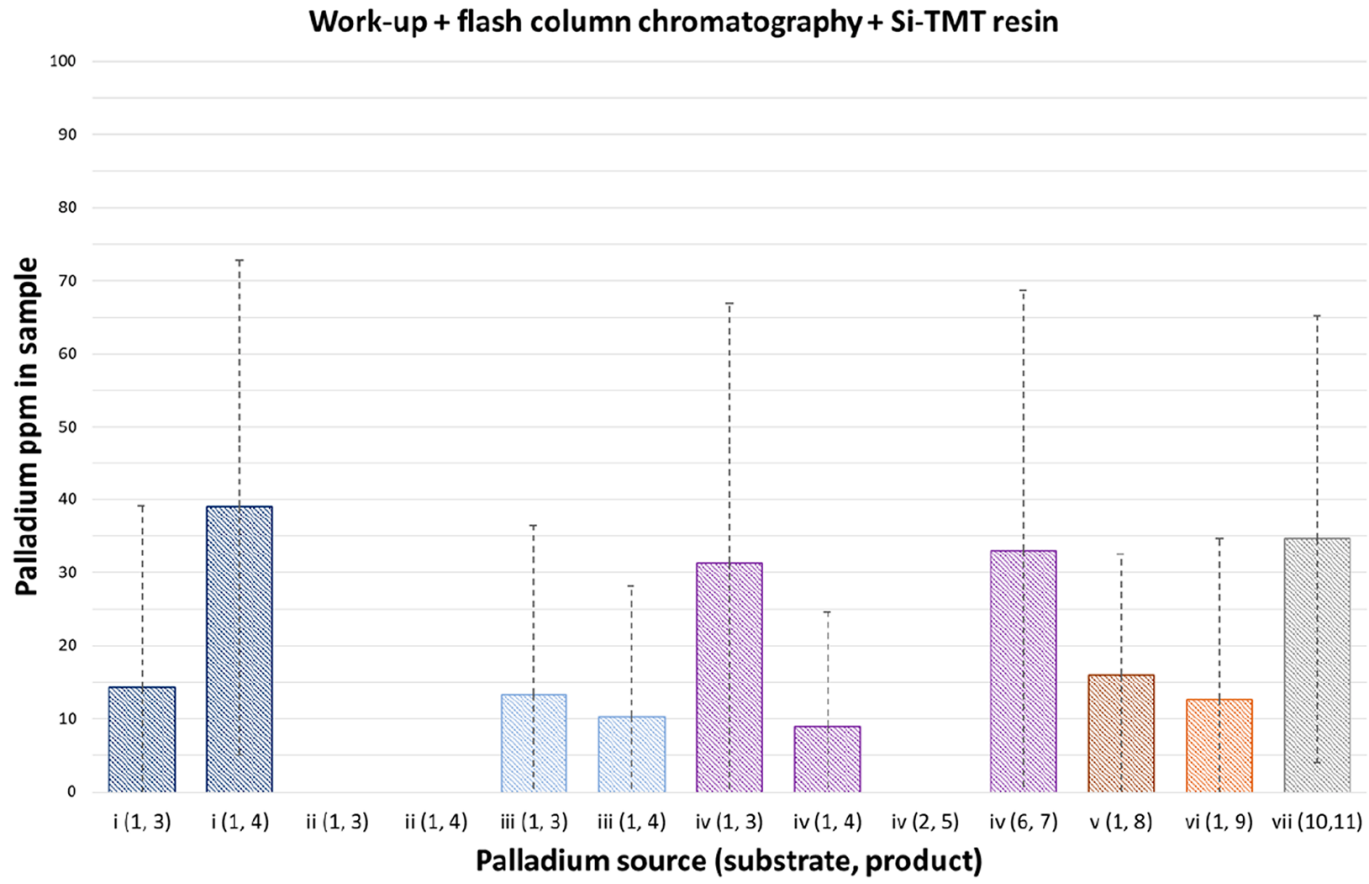
Residual palladium levels in reaction samples following automated (Biotage) flash chromatography and metal scavenging using Si-TMT resin (reaction *n* = 3; SD = dashed lines).

In line with other published data,^65,66^ the use of column chromatography followed by scavenging resin was found to be effective in removing residual palladium, with the average level in isolated samples for each reaction type/palladium source being found to be <50 ppm (Table S5C). Overall, 5/39 products had residual palladium levels above 50 ppm, and all five of these samples had levels below 100 ppm. Three Suzuki–Miyaura reaction conditions afforded products with palladium levels below the limit of detection: ii(1,3), ii(1,4), and iv(2,5).

The use of the scavenging resin as a final purification step was therefore shown to be reliable and effective in reducing the level of residual palladium in reaction products to <100 ppm under all of the conditions evaluated.

In terms of the effectiveness of chromatography and resin purification to remove palladium from crude products, we found that on average, using column chromatography followed by resin purification removed ∼98% of the residual palladium compared with the crude sample (Table S3B).

For the first time, a systematic study has been undertaken, with the objective being to quantify contamination in reaction products arising from commonly employed metal-based reagents. For this pilot study, palladium was studied due to its wide use. A representative selection of reactions that exemplified the typical use of palladium in contemporary organic synthesis was included. For the cross-coupling reactions, multiple coupling partners containing different functional groups were selected. Each reaction was run in triplicate, by different chemists, to mimic the typical variation shown in undertaking the same reaction by different laboratories.

For Suzuki–Miyaura and Buchwald–Hartwig couplings (palladium sources (i)–(vi), Scheme 1), very high levels of residual palladium were observed in some reaction products, even following chromatographic purification (Figure 1A,B). This suggests that additional palladium scavenging techniques should be used to remove residual metal from samples to minimize the potential for problems in biological assays or subsequent synthetic steps.

Metal-catalyzed reduction (palladium source (vii), Scheme 1) typically resulted in low residual metal content throughout, with some crude products returning results below the limit of quantification (Figure 1C and Table S5A). This is likely due to the specific experimental procedure and the heterogeneous nature of the reagents.

Overall, our experiments demonstrate that flash column chromatography alone is insufficient to remove palladium impurities, with only 90% of residue eliminated on average compared with that in the pre-column crude sample. On the basis of these results, we suggest that there should be a greater utilization of metal scavenging reagents, which are readily available and cheap, to reduce residual palladium to sub-100 ppm levels. Using this additional step removes >98% of the residue on average compared with crude samples in our pilot study. Moreover, the routine use of a scavenging step should be considered if there is potential for interference by residual palladium in a subsequent synthetic step or biological assay.

Various additional observations were made during this pilot study and data analysis (Table S6).

This pilot study also shows that the absolute effectiveness of chromatography in removing palladium varies widely and unpredictably on a case-by-case basis. More frequent evidence of the quantification of residual metal levels in reaction products should therefore be obtained by medicinal chemists as part of their compound characterization data. We suggest that journal editors and reviewers should, where the synthetic methodology justifies it, request the provision of analytical data to demonstrate the removal of or quantify the residue arising from metal catalyst use, in particular, where scavenging resins have not been employed.

Although further studies are needed to define more clearly how metal contamination levels correlate with deleterious effects in representative biological assays, we suggest that 100 ppm should be considered as the maximum acceptable palladium level in a screening sample. This is readily achievable with only minor and low-cost amendments to typical synthetic procedures.

Overall, these data indicate that whereas column chromatography does remove residual palladium to below 100 ppm in over half of the reactions undertaken herein, on the basis of the complete data set, it is impossible to predict to which reaction conditions, reagents, or building blocks this success will apply. Accordingly, chemists cannot be certain that reaction products contain an acceptably low residual palladium level unless additional purification steps are routinely incorporated into synthetic practice or the more routine quantification of residual palladium levels is undertaken. In projects where there is a possibility of residual palladium interfering with biological assays or in subsequent reactions, measurement of the palladium content, and employment of palladium scavenging methods could minimise the potential for future problems.^67−73^

The following five guidelines are recommended for chemists using palladium-mediated reactions, in particular, medicinal chemists producing screening compounds for lead optimization programs:

1. Palladium scavenging reagents or procedures should be used following column chromatography purification and before the reaction product is used in a subsequent process or assay.
2. Quantification of residual palladium should be undertaken more routinely to verify the lack of metal contamination, in particular, for compounds used to make important project decisions. We suggest that the appropriate maximum level of palladium in reaction products destined for testing in biological assays of any type should be 100 ppm.
3. Consideration should be given to the use of reagent combinations or catalysts with potential for leaving lower impurity levels, such as encapsulated metals^69^ or catalysts with high turnover numbers (TONs).^70^
4. Evidence that residual palladium levels have been quantified and shown to be within acceptable limits should be provided for any compounds that have been synthesized using a reaction sequence where palladium-catalyzed reactions have been used in the final or penultimate step and for which biological screening data are being generated.
5. Journal editors should consider requiring authors to either provide appropriate trace-metal analytical data for compounds where it may impact the study as a whole or acknowledge that they have considered the potential impact of trace residues and deem it not to be a relevant factor for their work. Authors and readers of patent applications should similarly be aware of the potential impact of trace-metal impurities, including palladium, because their use is also prolific therein.^20^

This pilot study has provided data to support future, more expansive investigations. A comprehensive, systematic, and quantitative evaluation of residual trace impurities arising from the use of specific palladium reagents in heterogeneous or homogeneous reactions is warranted in the first instance, including the study of further reaction classes (e.g., Heck, Stille, and Sonogashira reactions). Extension to other commonly used metals in organic synthesis, including zinc, platinum, iron, copper, nickel, ruthenium, and rhodium, is also justified. We consider it important to evaluate other purification methods, including HPLC (high-performance liquid chromatography) and SFC (supercritical fluid chromatography), which are widely used techniques in industry and elsewhere, along with alternative scavenging techniques. Finally, further studies to provide detailed support for our suggested 100 ppm “maximum” level of metal impurity using a representative range of biochemical and cellular assay systems are desirable.

## Experimental Procedures

The experimental protocols, workup, and isolation procedures for all reactions were standardized during this study and were based on described procedures. (See the Supporting Information.) Reactions were conducted by different chemists drawn from a team of six, each having ∼5+ years of synthetic experience following a chemistry degree or equivalent to best mimic real-world project conditions.

In total, 39 individual reactions were used to generate the data for this preliminary study. ICP-MS analysis was performed on samples at three different points in the workup/purification process to determine the levels of residual palladium (“crude”, “column”, and “resin”; see the Supporting Information).^71,72^

## Supporting Information Available

Literature analysis, tables summarizing Pd quantification levels using various purification techniques, detailed synthetic procedures, compound characterization, and spectral data (PDF)
