# Supplementary material for: Pilot Study to Quantify Palladium Impurities in Lead-like Compounds Following Commonly Used Purification Techniques
Source: ACS Med Chem Lett. 2022 Jan 20;13(2):262–70. doi: 10.1021/acsmedchemlett.1c00638 (PMC8842129; doi:10.1021/acsmedchemlett.1c00638)
Supplement: Supplementary file 1 — ml1c00638_si_001.pdf [file ml1c00638_si_001.pdf]

**SUPPORTING INFORMATION**

# A pilot study to quantify palladium impurities in lead-like compounds following commonly used purification techniques

*Maria Chatzopoulou[1#], Katrina S. Madden[1#], Liam J. Bromhead[1], Christopher Greaves[2], Thomas J. Cogswell[2], Solange Da Silva Pinto[2], Sébastien R. G. Galan[1], Irene Georgiou[2], Matthew S. Kennedy[2], Alice Kennett[1], Geraint Apps[3], Angela J. Russell[1,2], and Graham M. Wynne[1,4]**

[1] Department of Chemistry, Chemistry Research Laboratory, University of Oxford, Oxford, OX1 3TA, United Kingdom
[2] Department of Pharmacology, University of Oxford, Mansfield Road, Oxford, OX1 3PQ, United Kingdom
[3] CEMAS, Imperial House, Oaklands Business Centre, Oaklands Park, Wokingham, Berkshire, RG41 2FD, United Kingdom
[4] OxStem Limited, Midland House, West Way, Botley, Oxford, England, OX2 0PH, United Kingdom
[#] *these authors contributed equally*
[*] *Corresponding author, current email address: graham.wynne@930consulting.co.uk*

**Table of Contents** **Page**

| | |
|---|---|
| Literature Analysis | S2 |
| Table S1 | S3 |
| Table S2 | S4 – S13 |
| Tables S3A-B | S14 |
| Tables S4A-C | S15 |
| Tables S5A-C | S16 – S17 |
| Table S6 | S17 |
| General experimental, abbreviations, synthetic & purification procedures | S18 – S23 |
| Spectra | S24 – S39 |

Literature Analysis

To provide some baseline data on the extent to which palladium has been used in pre-clinical drug discovery projects and establish the frequency with which its removal from reaction mixtures was quantified, we used Reaxys (https://www.reaxys.com) to identify and categorise reactions in six major organic and medicinal chemistry journals during the year 2018. The selected journals were Journal of Organic Chemistry, Journal of Medicinal Chemistry (American Chemical Society); European Journal of Medicinal Chemistry, Bioorganic & Medicinal Chemistry, Bioorganic and Medicinal Chemistry Letters (Elsevier) and Organic and Biomolecular Chemistry (Royal Society of Chemistry).

From over half a million reactions found, more than half used some form of metal (or metalloid) catalysed reaction (258713 from 545404, *Table S1*). Of these, the metal most frequently used was palladium, which was employed in 24% of metal-based synthetic transformations (*Figure S1*). Copper was the next most often used, in 12% of cases.

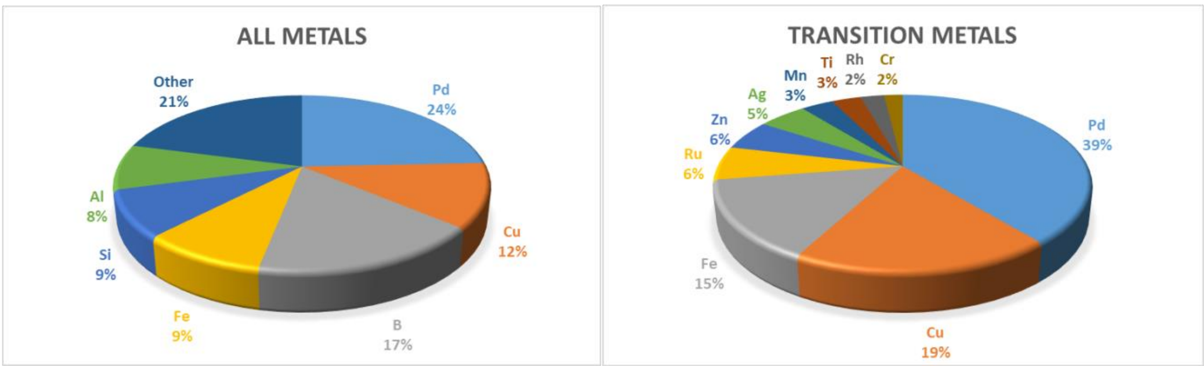


*Figure S1. Palladium is the most frequently used metal in synthetic procedures reported in representative organic and medicinal chemistry journals in 2018*

To probe this further, PubMed was used to retrieve digital object identifiers (DOI) from the list of papers. Sixty papers were randomly selected from each journal, giving a sample size of 360 papers. Each paper and its experimental section were reviewed in detail for whether the authors had used a palladium-catalysed reaction, and if so, whether residual palladium was quantified at any point, or whether a scavenger resin or some other chelation-based workup was employed and reported.

From the 360 papers sampled in total, 112 (31%) used palladium in one or more synthetic steps. Of the 112 papers, only 2 (<2% of total sample) quantified the palladium within the resulting compound(s). One of these (https://doi.org/10.1021/acs.joc.8b02269) was found to be an article from an industrial process development / scale-up group, and whilst the other (https://doi.org/10.1021/acs.jmedchem.8b01258) did indeed quantify palladium in a small (6/26) subset of compounds prepared for biological testing, a rationale was not provided for undertaking the analysis itself, or why only selected compounds were tested. Similarly, only a very few procedures used any form of scavenging reagent in purification of the final reaction product (*Table S2).*

Table S1. Usage of metals (or metalloids) in synthetic procedures in 6 major organic and medicinal chemistry journals during the year 2018 (reactions identified and categorised by Reaxys, https://www.reaxys.com)

| Journal | Reaction No | Catalyst classes - active center catalysts | Pd* | Cu* | B | Fe* | Si | Al | Ru* | Zn* | Ag* | Sn | Mn* | Ti* | Ce | Rh* | Cr* |
|---|---|---|---|---|---|---|---|---|---|---|---|---|---|---|---|---|---|
| JOC | 117381 | 66019 | 18913 | 14399 | 12143 | 4713 | 12194 | 10460 | 7574 | 3865 | 6189 | 1493 | 1739 | 2189 | 2609 | 3074 | 1406 |
| JMC | 142368 | 74883 | 34263 | 10923 | 24278 | 13174 | 6239 | 8171 | 3185 | 3402 | 1505 | 2245 | 2830 | 2911 | 954 | 473 | 779 |
| EJMC | 104292 | 38928 | 12400 | 5434 | 9537 | 6754 | 3981 | 4294 | 565 | 1477 | 673 | 1499 | 1365 | 660 | 1095 | 111 | 910 |
| OBC | 62635 | 30443 | 8862 | 8360 | 6211 | 1606 | 3879 | 3527 | 2027 | 1674 | 2588 | 1125 | 751 | 969 | 923 | 1746 | 578 |
| BMC | 58256 | 23222 | 7189 | 2717 | 5451 | 3615 | 3277 | 2688 | 488 | 1664 | 687 | 763 | 572 | 172 | 195 | 375 | 310 |
| BMCL | 60472 | 25218 | 11356 | 3262 | 7287 | 5121 | 3022 | 2877 | 874 | 1107 | 318 | 996 | 742 | 305 | 293 | 85 | 603 |
| *SUM:* | 545404 | 258713 | 92983 | 45095 | 64907 | 34983 | 32592 | 32017 | 14713 | 13189 | 11960 | 8121 | 7999 | 7206 | 6069 | 5864 | 4586 |

* transition metals, JOC: Journal of Organic Chemistry, JMC: Journal of Medicinal Chemistry, EJMC: European Journal of Medicinal Chemistry, OBC: Organic and Biomolecular Chemistry, BMC: Bioorganic & Medicinal Chemistry, BMCL: Bioorganic & Medicinal Chemistry Letters

Table S2. Literature analysis for palladium use, scavenging and quantification from 360 papers during 2018

| DOI | Journal | Synthesis | Pd used | Scavenger or chelator | Pd quant |
|---|---|---|---|---|---|
| https://dx.doi.org/10.1016/j.bmc.2017.03.067 | BMC | 1 | 0 | 0 | |
| https://dx.doi.org/10.1016/j.bmc.2017.07.012 | BMC | 1 | 0 | 0 | |
| https://dx.doi.org/10.1016/j.bmc.2017.08.028 | BMC | 1 | 0 | 0 | |
| https://dx.doi.org/10.1016/j.bmc.2017.09.016 | BMC | 1 | 0 | 0 | |
| https://dx.doi.org/10.1016/j.bmc.2017.09.019 | BMC | 1 | 1 | 0 | 0 |
| https://dx.doi.org/10.1016/j.bmc.2017.11.017 | BMC | 0 | 0 | 0 | |
| https://dx.doi.org/10.1016/j.bmc.2017.11.022 | BMC | 1 | 1 | 0 | 0 |
| https://dx.doi.org/10.1016/j.bmc.2017.11.026 | BMC | 0 | 0 | 0 | |
| https://dx.doi.org/10.1016/j.bmc.2017.11.027 | BMC | 1 | 0 | 0 | |
| https://dx.doi.org/10.1016/j.bmc.2017.11.028 | BMC | 1 | 0 | 0 | |
| https://dx.doi.org/10.1016/j.bmc.2017.11.030 | BMC | 1 | 0 | 0 | |
| https://dx.doi.org/10.1016/j.bmc.2017.11.033 | BMC | 1 | 0 | 0 | |
| https://dx.doi.org/10.1016/j.bmc.2017.11.036 | BMC | 1 | 1 | 0 | 0 |
| https://dx.doi.org/10.1016/j.bmc.2017.11.051 | BMC | 1 | 0 | 0 | |
| https://dx.doi.org/10.1016/j.bmc.2017.12.011 | BMC | 1 | 0 | 0 | |
| https://dx.doi.org/10.1016/j.bmc.2017.12.013 | BMC | 1 | 0 | 0 | |
| https://dx.doi.org/10.1016/j.bmc.2017.12.022 | BMC | 1 | 0 | 0 | |
| https://dx.doi.org/10.1016/j.bmc.2017.12.023 | BMC | 1 | 1 | 0 | 0 |
| https://dx.doi.org/10.1016/j.bmc.2017.12.030 | BMC | 1 | 1 | 0 | 0 |
| https://dx.doi.org/10.1016/j.bmc.2017.12.035 | BMC | 1 | 0 | 0 | |
| https://dx.doi.org/10.1016/j.bmc.2017.12.040 | BMC | 1 | 0 | 0 | |
| https://dx.doi.org/10.1016/j.bmc.2017.12.047 | BMC | 1 | 0 | 0 | |
| https://dx.doi.org/10.1016/j.bmc.2018.02.014 | BMC | 1 | 0 | 0 | |
| https://dx.doi.org/10.1016/j.bmc.2018.02.017 | BMC | 1 | 1 | 0 | 0 |
| https://dx.doi.org/10.1016/j.bmc.2018.02.025 | BMC | 1 | 0 | 0 | |
| https://dx.doi.org/10.1016/j.bmc.2018.02.032 | BMC | 1 | 1 | 0 | 0 |
| https://dx.doi.org/10.1016/j.bmc.2018.02.036 | BMC | 1 | 1 | 0 | 0 |
| https://dx.doi.org/10.1016/j.bmc.2018.02.042 | BMC | 1 | 1 | 0 | 0 |
| https://dx.doi.org/10.1016/j.bmc.2018.02.051 | BMC | 1 | 0 | 0 | |
| https://dx.doi.org/10.1016/j.bmc.2018.03.035 | BMC | 1 | 0 | 0 | |
| https://dx.doi.org/10.1016/j.bmc.2018.04.020 | BMC | 1 | 0 | 0 | |
| https://dx.doi.org/10.1016/j.bmc.2018.04.038 | BMC | 1 | 0 | 0 | |
| https://dx.doi.org/10.1016/j.bmc.2018.04.049 | BMC | 1 | 0 | 0 | |
| https://dx.doi.org/10.1016/j.bmc.2018.04.051 | BMC | 1 | 1 | 0 | 0 |
| https://dx.doi.org/10.1016/j.bmc.2018.05.002 | BMC | 1 | 0 | 0 | |

| DOI | Journal | Synthesis | Pd used | Scavenger or chelator | Pd quant |
|---|---|---|---|---|---|
| https://dx.doi.org/10.1016/j.bmc.2018.05.023 | BMC | 1 | 0 | 0 | |
| https://dx.doi.org/10.1016/j.bmc.2018.05.050 | BMC | 1 | 0 | 0 | |
| https://dx.doi.org/10.1016/j.bmc.2018.06.001 | BMC | 1 | 1 | 0 | 0 |
| https://dx.doi.org/10.1016/j.bmc.2018.06.003 | BMC | 1 | 1 | 0 | 0 |
| https://dx.doi.org/10.1016/j.bmc.2018.06.006 | BMC | 1 | 1 | 0 | 0 |
| https://dx.doi.org/10.1016/j.bmc.2018.06.009 | BMC | 0 | 0 | 0 | |
| https://dx.doi.org/10.1016/j.bmc.2018.07.002 | BMC | 0 | 0 | 0 | |
| https://dx.doi.org/10.1016/j.bmc.2018.07.006 | BMC | 1 | 0 | 0 | 0 |
| https://dx.doi.org/10.1016/j.bmc.2018.07.025 | BMC | 1 | 0 | 0 | |
| https://dx.doi.org/10.1016/j.bmc.2018.07.026 | BMC | 1 | 1 | 0 | 0 |
| https://dx.doi.org/10.1016/j.bmc.2018.07.027 | BMC | 1 | 0 | 0 | |
| https://dx.doi.org/10.1016/j.bmc.2018.07.031 | BMC | 0 | 0 | 0 | |
| https://dx.doi.org/10.1016/j.bmc.2018.07.041 | BMC | 1 | 0 | 0 | 0 |
| https://dx.doi.org/10.1016/j.bmc.2018.07.046 | BMC | 1 | 0 | 0 | 0 |
| https://dx.doi.org/10.1016/j.bmc.2018.08.013 | BMC | 1 | 0 | 0 | 0 |
| https://dx.doi.org/10.1016/j.bmc.2018.08.016 | BMC | 1 | 0 | 0 | |
| https://dx.doi.org/10.1016/j.bmc.2018.08.018 | BMC | 1 | 0 | 0 | 0 |
| https://dx.doi.org/10.1016/j.bmc.2018.08.033 | BMC | 1 | 0 | 0 | 0 |
| https://dx.doi.org/10.1016/j.bmc.2018.10.036 | BMC | 1 | 0 | 0 | 0 |
| https://dx.doi.org/10.1016/j.bmc.2018.11.013 | BMC | 1 | 0 | 0 | 0 |
| https://dx.doi.org/10.1016/j.bmc.2018.11.021 | BMC | 1 | 0 | 0 | |
| https://dx.doi.org/10.1016/j.bmc.2018.11.039 | BMC | 1 | 1 | 0 | 0 |
| https://dx.doi.org/10.1016/j.bmc.2018.12.040 | BMC | 1 | 0 | 0 | 0 |
| https://dx.doi.org/10.1016/j.bmc.2018.12.041 | BMC | 1 | 0 | 0 | 0 |
| https://dx.doi.org/10.1016/j.bmc.2018.12.042 | BMC | 1 | 0 | 0 | |
| https://dx.doi.org/10.1016/j.bmcl.2017.11.021 | BMCL | 1 | 0 | 0 | |
| https://dx.doi.org/10.1016/j.bmcl.2017.11.026 | BMCL | 1 | 1 | 0 | 0 |
| https://dx.doi.org/10.1016/j.bmcl.2017.11.031 | BMCL | 1 | 0 | 0 | |
| https://dx.doi.org/10.1016/j.bmcl.2017.11.033 | BMCL | 0 | 0 | 0 | |
| https://dx.doi.org/10.1016/j.bmcl.2017.11.048 | BMCL | 1 | 1 | 0 | 0 |
| https://dx.doi.org/10.1016/j.bmcl.2017.12.011 | BMCL | 1 | 1 | 0 | 0 |
| https://dx.doi.org/10.1016/j.bmcl.2017.12.012 | BMCL | 1 | 0 | 0 | |
| https://dx.doi.org/10.1016/j.bmcl.2017.12.031 | BMCL | 0 | 0 | 0 | |
| https://dx.doi.org/10.1016/j.bmcl.2017.12.045 | BMCL | 1 | 0 | 0 | |
| https://dx.doi.org/10.1016/j.bmcl.2017.12.054 | BMCL | 1 | 1 | 0 | 0 |
| https://dx.doi.org/10.1016/j.bmcl.2017.12.064 | BMCL | 1 | 0 | 0 | |
| https://dx.doi.org/10.1016/j.bmcl.2018.01.005 | BMCL | 1 | 0 | 0 | |
| https://dx.doi.org/10.1016/j.bmcl.2018.01.023 | BMCL | 1 | 0 | 0 | |
| https://dx.doi.org/10.1016/j.bmcl.2018.01.042 | BMCL | 1 | 0 | 0 | |
| https://dx.doi.org/10.1016/j.bmcl.2018.01.051 | BMCL | 0 | 0 | 0 | |

| DOI | Journal | Synthesis | Pd used | Scavenger or chelator | Pd quant |
|---|---|---|---|---|---|
| https://dx.doi.org/10.1016/j.bmcl.2018.01.064 | BMCL | 1 | 0 | 0 | |
| https://dx.doi.org/10.1016/j.bmcl.2018.02.016 | BMCL | 0 | 0 | 0 | |
| https://dx.doi.org/10.1016/j.bmcl.2018.02.027 | BMCL | 1 | 1 | 0 | 0 |
| https://dx.doi.org/10.1016/j.bmcl.2018.02.028 | BMCL | 1 | 0 | 0 | |
| https://dx.doi.org/10.1016/j.bmcl.2018.02.030 | BMCL | 1 | 0 | 0 | |
| https://dx.doi.org/10.1016/j.bmcl.2018.02.032 | BMCL | 1 | 0 | 0 | |
| https://dx.doi.org/10.1016/j.bmcl.2018.03.007 | BMCL | 1 | 0 | 0 | |
| https://dx.doi.org/10.1016/j.bmcl.2018.03.024 | BMCL | 1 | 0 | 0 | |
| https://dx.doi.org/10.1016/j.bmcl.2018.03.062 | BMCL | 1 | 0 | 0 | |
| https://dx.doi.org/10.1016/j.bmcl.2018.03.064 | BMCL | 0 | 0 | 0 | |
| https://dx.doi.org/10.1016/j.bmcl.2018.03.066 | BMCL | 1 | 0 | 0 | |
| https://dx.doi.org/10.1016/j.bmcl.2018.03.074 | BMCL | 1 | 0 | 0 | |
| https://dx.doi.org/10.1016/j.bmcl.2018.04.004 | BMCL | 1 | 1 | 0 | 0 |
| https://dx.doi.org/10.1016/j.bmcl.2018.04.020 | BMCL | 1 | 1 | 0 | 0 |
| https://dx.doi.org/10.1016/j.bmcl.2018.04.044 | BMCL | 1 | 1 | 0 | 0 |
| https://dx.doi.org/10.1016/j.bmcl.2018.04.052 | BMCL | 0 | 0 | 0 | |
| https://dx.doi.org/10.1016/j.bmcl.2018.05.010 | BMCL | 1 | 0 | 0 | |
| https://dx.doi.org/10.1016/j.bmcl.2018.06.018 | BMCL | 1 | 1 | 0 | 0 |
| https://dx.doi.org/10.1016/j.bmcl.2018.06.023 | BMCL | 0 | 0 | 0 | |
| https://dx.doi.org/10.1016/j.bmcl.2018.06.028 | BMCL | 1 | 1 | 0 | 0 |
| https://dx.doi.org/10.1016/j.bmcl.2018.06.033 | BMCL | 1 | 0 | 0 | |
| https://dx.doi.org/10.1016/j.bmcl.2018.06.047 | BMCL | 1 | 1 | 0 | 0 |
| https://dx.doi.org/10.1016/j.bmcl.2018.07.021 | BMCL | 1 | 1 | 0 | 0 |
| https://dx.doi.org/10.1016/j.bmcl.2018.07.022 | BMCL | 0 | 0 | 0 | |
| https://dx.doi.org/10.1016/j.bmcl.2018.07.031 | BMCL | 1 | 0 | 0 | |
| https://dx.doi.org/10.1016/j.bmcl.2018.07.038 | BMCL | 1 | 0 | 0 | |
| https://dx.doi.org/10.1016/j.bmcl.2018.08.024 | BMCL | 1 | 1 | 0 | 0 |
| https://dx.doi.org/10.1016/j.bmcl.2018.08.035 | BMCL | 1 | 0 | 0 | |
| https://dx.doi.org/10.1016/j.bmcl.2018.09.008 | BMCL | 1 | 0 | 0 | |
| https://dx.doi.org/10.1016/j.bmcl.2018.09.014 | BMCL | 1 | 0 | 0 | |
| https://dx.doi.org/10.1016/j.bmcl.2018.09.015 | BMCL | 1 | 0 | 0 | |
| https://dx.doi.org/10.1016/j.bmcl.2018.09.018 | BMCL | 1 | 0 | 0 | |
| https://dx.doi.org/10.1016/j.bmcl.2018.09.029 | BMCL | 1 | 0 | 0 | |
| https://dx.doi.org/10.1016/j.bmcl.2018.10.013 | BMCL | 1 | 0 | 0 | |
| https://dx.doi.org/10.1016/j.bmcl.2018.10.020 | BMCL | 1 | 0 | 0 | |
| https://dx.doi.org/10.1016/j.bmcl.2018.10.035 | BMCL | 1 | 0 | 0 | |
| https://dx.doi.org/10.1016/j.bmcl.2018.10.047 | BMCL | 1 | 0 | 0 | |
| https://dx.doi.org/10.1016/j.bmcl.2018.10.050 | BMCL | 1 | 1 | 0 | 0 |
| https://dx.doi.org/10.1016/j.bmcl.2018.11.012 | BMCL | 0 | 0 | 0 | |
| https://dx.doi.org/10.1016/j.bmcl.2018.12.005 | BMCL | 1 | 0 | 0 | |

| DOI | Journal | Synthesis | Pd used | Scavenger or chelator | Pd quant |
|---|---|---|---|---|---|
| https://dx.doi.org/10.1016/j.bmcl.2018.12.009 | BMCL | 1 | 0 | 0 | |
| https://dx.doi.org/10.1016/j.bmcl.2018.12.018 | BMCL | 1 | 0 | 0 | |
| https://dx.doi.org/10.1016/j.bmcl.2018.12.031 | BMCL | 1 | 0 | 0 | |
| https://dx.doi.org/10.1016/j.bmcl.2018.12.040 | BMCL | 1 | 1 | 0 | 0 |
| https://dx.doi.org/10.1016/j.bmcl.2018.12.042 | BMCL | 1 | 1 | 0 | 0 |
| https://dx.doi.org/10.1016/j.ejmech.2017.10.033 | EJMC | 1 | 1 | 0 | 0 |
| https://dx.doi.org/10.1016/j.ejmech.2017.10.067 | EJMC | 1 | 0 | 0 | |
| https://dx.doi.org/10.1016/j.ejmech.2017.10.071 | EJMC | 1 | 0 | 0 | |
| https://dx.doi.org/10.1016/j.ejmech.2017.11.056 | EJMC | 1 | 0 | 0 | |
| https://dx.doi.org/10.1016/j.ejmech.2017.11.086 | EJMC | 1 | 0 | 0 | |
| https://dx.doi.org/10.1016/j.ejmech.2017.11.094 | EJMC | 1 | 0 | 0 | |
| https://dx.doi.org/10.1016/j.ejmech.2017.11.098 | EJMC | 1 | 0 | 0 | |
| https://dx.doi.org/10.1016/j.ejmech.2017.12.017 | EJMC | 1 | 0 | 0 | |
| https://dx.doi.org/10.1016/j.ejmech.2017.12.035 | EJMC | 1 | 1 | 0 | 0 |
| https://dx.doi.org/10.1016/j.ejmech.2017.12.050 | EJMC | 1 | 1 | 0 | 0 |
| https://dx.doi.org/10.1016/j.ejmech.2017.12.055 | EJMC | 1 | 0 | 0 | |
| https://dx.doi.org/10.1016/j.ejmech.2017.12.075 | EJMC | 1 | 0 | 0 | |
| https://dx.doi.org/10.1016/j.ejmech.2018.01.049 | EJMC | 1 | 1 | 0 | 0 |
| https://dx.doi.org/10.1016/j.ejmech.2018.01.064 | EJMC | 1 | 0 | 0 | |
| https://dx.doi.org/10.1016/j.ejmech.2018.01.086 | EJMC | 1 | 0 | 0 | |
| https://dx.doi.org/10.1016/j.ejmech.2018.02.005 | EJMC | 1 | 0 | 0 | |
| https://dx.doi.org/10.1016/j.ejmech.2018.02.055 | EJMC | 1 | 0 | 0 | |
| https://dx.doi.org/10.1016/j.ejmech.2018.02.076 | EJMC | 1 | 0 | 0 | |
| https://dx.doi.org/10.1016/j.ejmech.2018.03.004 | EJMC | 1 | 0 | 0 | |
| https://dx.doi.org/10.1016/j.ejmech.2018.03.005 | EJMC | 1 | 1 | 0 | 0 |
| https://dx.doi.org/10.1016/j.ejmech.2018.03.006 | EJMC | 1 | 1 | 0 | 0 |
| https://dx.doi.org/10.1016/j.ejmech.2018.03.022 | EJMC | 1 | 0 | 0 | |
| https://dx.doi.org/10.1016/j.ejmech.2018.04.058 | EJMC | 1 | 0 | 0 | |
| https://dx.doi.org/10.1016/j.ejmech.2018.04.059 | EJMC | 1 | 0 | 0 | |
| https://dx.doi.org/10.1016/j.ejmech.2018.05.027 | EJMC | 1 | 1 | 0 | 0 |
| https://dx.doi.org/10.1016/j.ejmech.2018.05.050 | EJMC | 1 | 0 | 0 | |
| https://dx.doi.org/10.1016/j.ejmech.2018.05.051 | EJMC | 1 | 1 | 0 | 0 |
| https://dx.doi.org/10.1016/j.ejmech.2018.05.056 | EJMC | 1 | 0 | 0 | |
| https://dx.doi.org/10.1016/j.ejmech.2018.06.001 | EJMC | 1 | 0 | 0 | |
| https://dx.doi.org/10.1016/j.ejmech.2018.06.012 | EJMC | 1 | 1 | 0 | 0 |
| https://dx.doi.org/10.1016/j.ejmech.2018.06.026 | EJMC | 1 | 0 | 0 | |
| https://dx.doi.org/10.1016/j.ejmech.2018.06.060 | EJMC | 1 | 1 | 0 | 0 |
| https://dx.doi.org/10.1016/j.ejmech.2018.07.002 | EJMC | 1 | 0 | 0 | |
| https://dx.doi.org/10.1016/j.ejmech.2018.07.058 | EJMC | 1 | 0 | 0 | |
| https://dx.doi.org/10.1016/j.ejmech.2018.07.066 | EJMC | 1 | 0 | 0 | |

| DOI | Journal | Synthesis | Pd used | Scavenger or chelator | Pd quant |
|---|---|---|---|---|---|
| https://dx.doi.org/10.1016/j.ejmech.2018.08.019 | EJMC | 1 | 1 | 0 | 0 |
| https://dx.doi.org/10.1016/j.ejmech.2018.08.067 | EJMC | 1 | 0 | 0 | |
| https://dx.doi.org/10.1016/j.ejmech.2018.08.081 | EJMC | 1 | 0 | 0 | |
| https://dx.doi.org/10.1016/j.ejmech.2018.08.090 | EJMC | 1 | 1 | 0 | 0 |
| https://dx.doi.org/10.1016/j.ejmech.2018.09.003 | EJMC | 1 | 1 | 0 | 0 |
| https://dx.doi.org/10.1016/j.ejmech.2018.09.004 | EJMC | 1 | 1 | 0 | 0 |
| https://dx.doi.org/10.1016/j.ejmech.2018.09.034 | EJMC | 1 | 0 | 0 | |
| https://dx.doi.org/10.1016/j.ejmech.2018.09.062 | EJMC | 1 | 0 | 0 | |
| https://dx.doi.org/10.1016/j.ejmech.2018.10.011 | EJMC | 1 | 0 | 0 | |
| https://dx.doi.org/10.1016/j.ejmech.2018.10.020 | EJMC | 1 | 0 | 0 | |
| https://dx.doi.org/10.1016/j.ejmech.2018.10.037 | EJMC | 1 | 0 | 0 | |
| https://dx.doi.org/10.1016/j.ejmech.2018.10.046 | EJMC | 1 | 0 | 0 | |
| https://dx.doi.org/10.1016/j.ejmech.2018.10.048 | EJMC | 1 | 1 | 0 | 0 |
| https://dx.doi.org/10.1016/j.ejmech.2018.11.006 | EJMC | 1 | 0 | 0 | |
| https://dx.doi.org/10.1016/j.ejmech.2018.11.018 | EJMC | 1 | 0 | 0 | |
| https://dx.doi.org/10.1016/j.ejmech.2018.11.019 | EJMC | 1 | 1 | 0 | 0 |
| https://dx.doi.org/10.1016/j.ejmech.2018.11.048 | EJMC | 1 | 0 | 0 | |
| https://dx.doi.org/10.1016/j.ejmech.2018.11.049 | EJMC | 1 | 0 | 0 | |
| https://dx.doi.org/10.1016/j.ejmech.2018.11.050 | EJMC | 1 | 1 | 0 | 0 |
| https://dx.doi.org/10.1016/j.ejmech.2018.11.051 | EJMC | 1 | 1 | 0 | 0 |
| https://dx.doi.org/10.1016/j.ejmech.2018.12.008 | EJMC | 1 | 1 | 0 | 0 |
| https://dx.doi.org/10.1016/j.ejmech.2018.12.030 | EJMC | 1 | 0 | 0 | |
| https://dx.doi.org/10.1016/j.ejmech.2018.12.033 | EJMC | 1 | 0 | 0 | |
| https://dx.doi.org/10.1016/j.ejmech.2018.12.067 | EJMC | 1 | 1 | 0 | 0 |
| https://dx.doi.org/10.1016/j.ejmech.2018.12.073 | EJMC | 1 | 0 | 0 | |
| https://dx.doi.org/10.1021/acs.jmedchem.7b00404 | JMC | 0 | 0 | 0 | |
| https://dx.doi.org/10.1021/acs.jmedchem.7b00530 | JMC | 1 | 0 | 0 | |
| https://dx.doi.org/10.1021/acs.jmedchem.7b00574 | JMC | 0 | 0 | 0 | |
| https://dx.doi.org/10.1021/acs.jmedchem.7b00675 | JMC | 1 | 0 | 0 | |
| https://dx.doi.org/10.1021/acs.jmedchem.7b01012 | JMC | 1 | 0 | 0 | |
| https://dx.doi.org/10.1021/acs.jmedchem.7b01051 | JMC | 1 | 0 | 0 | |
| https://dx.doi.org/10.1021/acs.jmedchem.7b01052 | JMC | 1 | 0 | 0 | |
| https://dx.doi.org/10.1021/acs.jmedchem.7b01091 | JMC | 1 | 1 | 0 | 0 |
| https://dx.doi.org/10.1021/acs.jmedchem.7b01138 | JMC | 1 | 0 | 0 | |
| https://dx.doi.org/10.1021/acs.jmedchem.7b01404 | JMC | 1 | 1 | 0 | 0 |
| https://dx.doi.org/10.1021/acs.jmedchem.7b01413 | JMC | 0 | 0 | 0 | |
| https://dx.doi.org/10.1021/acs.jmedchem.7b01417 | JMC | 1 | 1 | 0 | 0 |
| https://dx.doi.org/10.1021/acs.jmedchem.7b01445 | JMC | 0 | 0 | 0 | |
| https://dx.doi.org/10.1021/acs.jmedchem.7b01468 | JMC | 1 | 1 | 0 | 0 |
| https://dx.doi.org/10.1021/acs.jmedchem.7b01474 | JMC | 1 | 1 | 0 | 0 |

| DOI | Journal | Synthesis | Pd used | Scavenger or chelator | Pd quant |
|---|---|---|---|---|---|
| https://dx.doi.org/10.1021/acs.jmedchem.7b01547 | JMC | 1 | 1 | 0 | 0 |
| https://dx.doi.org/10.1021/acs.jmedchem.7b01581 | JMC | 1 | 1 | 0 | 0 |
| https://dx.doi.org/10.1021/acs.jmedchem.7b01684 | JMC | 0 | 0 | 0 | |
| https://dx.doi.org/10.1021/acs.jmedchem.7b01691 | JMC | 1 | 1 | 0 | 0 |
| https://dx.doi.org/10.1021/acs.jmedchem.7b01716 | JMC | 1 | 1 | 1 | 0 |
| https://dx.doi.org/10.1021/acs.jmedchem.7b01722 | JMC | 1 | 0 | 0 | |
| https://dx.doi.org/10.1021/acs.jmedchem.7b01746 | JMC | 1 | 0 | 0 | |
| https://dx.doi.org/10.1021/acs.jmedchem.7b01781 | JMC | 1 | 0 | 0 | |
| https://dx.doi.org/10.1021/acs.jmedchem.7b01830 | JMC | 1 | 0 | 0 | |
| https://dx.doi.org/10.1021/acs.jmedchem.7b01834 | JMC | 1 | 1 | 0 | 0 |
| https://dx.doi.org/10.1021/acs.jmedchem.7b01844 | JMC | 0 | 0 | 0 | |
| https://dx.doi.org/10.1021/acs.jmedchem.7b01863 | JMC | 1 | 0 | 0 | |
| https://dx.doi.org/10.1021/acs.jmedchem.7b01896 | JMC | 1 | 1 | 0 | 0 |
| https://dx.doi.org/10.1021/acs.jmedchem.8b00026 | JMC | 1 | 0 | 0 | |
| https://dx.doi.org/10.1021/acs.jmedchem.8b00036 | JMC | 1 | 0 | 0 | |
| https://dx.doi.org/10.1021/acs.jmedchem.8b00040 | JMC | 1 | 1 | 0 | 0 |
| https://dx.doi.org/10.1021/acs.jmedchem.8b00052 | JMC | 1 | 1 | 0 | 0 |
| https://dx.doi.org/10.1021/acs.jmedchem.8b00067 | JMC | 1 | 1 | 0 | 0 |
| https://dx.doi.org/10.1021/acs.jmedchem.8b00081 | JMC | 1 | 0 | 0 | |
| https://dx.doi.org/10.1021/acs.jmedchem.8b00099 | JMC | 1 | 0 | 0 | |
| https://dx.doi.org/10.1021/acs.jmedchem.8b00116 | JMC | 1 | 1 | 0 | 0 |
| https://dx.doi.org/10.1021/acs.jmedchem.8b00270 | JMC | 1 | 1 | 1 | 0 |
| https://dx.doi.org/10.1021/acs.jmedchem.8b00294 | JMC | 0 | 0 | 0 | |
| https://dx.doi.org/10.1021/acs.jmedchem.8b00305 | JMC | 1 | 1 | 0 | 0 |
| https://dx.doi.org/10.1021/acs.jmedchem.8b00403 | JMC | 1 | 0 | 0 | |
| https://dx.doi.org/10.1021/acs.jmedchem.8b00502 | JMC | 1 | 0 | 0 | |
| https://dx.doi.org/10.1021/acs.jmedchem.8b00557 | JMC | 1 | 1 | 0 | 0 |
| https://dx.doi.org/10.1021/acs.jmedchem.8b00639 | JMC | 1 | 1 | 0 | 0 |
| https://dx.doi.org/10.1021/acs.jmedchem.8b00655 | JMC | 1 | 0 | 0 | |
| https://dx.doi.org/10.1021/acs.jmedchem.8b00817 | JMC | 1 | 0 | 0 | |
| https://dx.doi.org/10.1021/acs.jmedchem.8b00906 | JMC | 1 | 0 | 0 | |
| https://dx.doi.org/10.1021/acs.jmedchem.8b00917 | JMC | 1 | 1 | 0 | 0 |
| https://dx.doi.org/10.1021/acs.jmedchem.8b00936 | JMC | 1 | 1 | 0 | 0 |
| https://dx.doi.org/10.1021/acs.jmedchem.8b00951 | JMC | 1 | 1 | 0 | 0 |
| https://dx.doi.org/10.1021/acs.jmedchem.8b00989 | JMC | 1 | 0 | 0 | |
| https://dx.doi.org/10.1021/acs.jmedchem.8b01023 | JMC | 1 | 1 | 0 | 0 |
| https://dx.doi.org/10.1021/acs.jmedchem.8b01219 | JMC | 1 | 0 | 0 | |
| https://dx.doi.org/10.1021/acs.jmedchem.8b01238 | JMC | 1 | 0 | 0 | |
| https://dx.doi.org/10.1021/acs.jmedchem.8b01258 | JMC | 1 | 1 | 0 | 1 |
| https://dx.doi.org/10.1021/acs.jmedchem.8b01343 | JMC | 1 | 0 | 0 | |

| DOI | Journal | Synthesis | Pd used | Scavenger or chelator | Pd quant |
|---|---|---|---|---|---|
| https://dx.doi.org/10.1021/acs.jmedchem.8b01397 | JMC | 0 | 0 | 0 | |
| https://dx.doi.org/10.1021/acs.jmedchem.8b01469 | JMC | 0 | 0 | 0 | |
| https://dx.doi.org/10.1021/acs.jmedchem.8b01497 | JMC | 1 | 1 | 0 | 0 |
| https://dx.doi.org/10.1021/acs.jmedchem.8b01755 | JMC | 1 | 1 | 0 | 0 |
| https://dx.doi.org/10.1021/acs.jmedchem.8b01769 | JMC | 1 | 1 | 0 | 0 |
| https://dx.doi.org/10.1021/acs.joc.7b02584 | JOC | 1 | 0 | 0 | |
| https://dx.doi.org/10.1021/acs.joc.7b02585 | JOC | 1 | 0 | 0 | |
| https://dx.doi.org/10.1021/acs.joc.7b02595 | JOC | 1 | 0 | 0 | |
| https://dx.doi.org/10.1021/acs.joc.7b02684 | JOC | 1 | 0 | 0 | |
| https://dx.doi.org/10.1021/acs.joc.7b02715 | JOC | 1 | 0 | 0 | |
| https://dx.doi.org/10.1021/acs.joc.7b02813 | JOC | 1 | 0 | 0 | |
| https://dx.doi.org/10.1021/acs.joc.7b02956 | JOC | 1 | 1 | 0 | 0 |
| https://dx.doi.org/10.1021/acs.joc.7b03022 | JOC | 1 | 0 | 0 | |
| https://dx.doi.org/10.1021/acs.joc.7b03080 | JOC | 1 | 0 | 0 | |
| https://dx.doi.org/10.1021/acs.joc.7b03119 | JOC | 1 | 0 | 0 | |
| https://dx.doi.org/10.1021/acs.joc.7b03150 | JOC | 1 | 0 | 0 | |
| https://dx.doi.org/10.1021/acs.joc.7b03223 | JOC | 1 | 0 | 0 | |
| https://dx.doi.org/10.1021/acs.joc.7b03268 | JOC | 1 | 1 | 0 | 0 |
| https://dx.doi.org/10.1021/acs.joc.7b03272 | JOC | 1 | 0 | 0 | |
| https://dx.doi.org/10.1021/acs.joc.8b00025 | JOC | 1 | 0 | 0 | |
| https://dx.doi.org/10.1021/acs.joc.8b00038 | JOC | 1 | 1 | 0 | 0 |
| https://dx.doi.org/10.1021/acs.joc.8b00052 | JOC | 1 | 1 | 0 | 0 |
| https://dx.doi.org/10.1021/acs.joc.8b00088 | JOC | 1 | 0 | 0 | |
| https://dx.doi.org/10.1021/acs.joc.8b00094 | JOC | 1 | 0 | 0 | |
| https://dx.doi.org/10.1021/acs.joc.8b00206 | JOC | 1 | 0 | 0 | |
| https://dx.doi.org/10.1021/acs.joc.8b00286 | JOC | 1 | 0 | 0 | |
| https://dx.doi.org/10.1021/acs.joc.8b00310 | JOC | 1 | 1 | 0 | 0 |
| https://dx.doi.org/10.1021/acs.joc.8b00412 | JOC | 1 | 1 | 0 | 0 |
| https://dx.doi.org/10.1021/acs.joc.8b00468 | JOC | 1 | 0 | 0 | |
| https://dx.doi.org/10.1021/acs.joc.8b00487 | JOC | 1 | 0 | 0 | |
| https://dx.doi.org/10.1021/acs.joc.8b00525 | JOC | 1 | 0 | 0 | |
| https://dx.doi.org/10.1021/acs.joc.8b00529 | JOC | 1 | 1 | 0 | 0 |
| https://dx.doi.org/10.1021/acs.joc.8b00585 | JOC | 1 | 1 | 0 | 0 |
| https://dx.doi.org/10.1021/acs.joc.8b00725 | JOC | 1 | 0 | 0 | |
| https://dx.doi.org/10.1021/acs.joc.8b00872 | JOC | 1 | 1 | 0 | 0 |
| https://dx.doi.org/10.1021/acs.joc.8b00970 | JOC | 1 | 0 | 0 | |
| https://dx.doi.org/10.1021/acs.joc.8b01020 | JOC | 1 | 0 | 0 | |
| https://dx.doi.org/10.1021/acs.joc.8b01042 | JOC | 1 | 0 | 0 | |
| https://dx.doi.org/10.1021/acs.joc.8b01218 | JOC | 1 | 1 | 0 | 0 |
| https://dx.doi.org/10.1021/acs.joc.8b01236 | JOC | 1 | 0 | 0 | |

| DOI | Journal | Synthesis | Pd used | Scavenger or chelator | Pd quant |
|---|---|---|---|---|---|
| https://dx.doi.org/10.1021/acs.joc.8b01252 | JOC | 1 | 0 | 0 | |
| https://dx.doi.org/10.1021/acs.joc.8b01266 | JOC | 1 | 0 | 0 | |
| https://dx.doi.org/10.1021/acs.joc.8b01273 | JOC | 1 | 0 | 0 | |
| https://dx.doi.org/10.1021/acs.joc.8b01276 | JOC | 1 | 0 | 0 | |
| https://dx.doi.org/10.1021/acs.joc.8b01316 | JOC | 1 | 0 | 0 | |
| https://dx.doi.org/10.1021/acs.joc.8b01362 | JOC | 1 | 1 | 0 | 0 |
| https://dx.doi.org/10.1021/acs.joc.8b01370 | JOC | 1 | 1 | 0 | 0 |
| https://dx.doi.org/10.1021/acs.joc.8b01377 | JOC | 1 | 0 | 0 | |
| https://dx.doi.org/10.1021/acs.joc.8b01405 | JOC | 1 | 1 | 0 | 0 |
| https://dx.doi.org/10.1021/acs.joc.8b01440 | JOC | 1 | 1 | 0 | 0 |
| https://dx.doi.org/10.1021/acs.joc.8b01446 | JOC | 1 | 1 | 0 | 0 |
| https://dx.doi.org/10.1021/acs.joc.8b01480 | JOC | 1 | 1 | 0 | 0 |
| https://dx.doi.org/10.1021/acs.joc.8b01567 | JOC | 1 | 0 | 0 | |
| https://dx.doi.org/10.1021/acs.joc.8b01582 | JOC | 1 | 1 | 0 | 0 |
| https://dx.doi.org/10.1021/acs.joc.8b01698 | JOC | 1 | 1 | 0 | 0 |
| https://dx.doi.org/10.1021/acs.joc.8b01887 | JOC | 1 | 0 | 0 | |
| https://dx.doi.org/10.1021/acs.joc.8b02076 | JOC | 1 | 1 | 0 | 0 |
| https://dx.doi.org/10.1021/acs.joc.8b02175 | JOC | 1 | 1 | 0 | 0 |
| https://dx.doi.org/10.1021/acs.joc.8b02208 | JOC | 1 | 0 | 0 | |
| https://dx.doi.org/10.1021/acs.joc.8b02269 | JOC | 1 | 1 | 1 | 1 |
| https://dx.doi.org/10.1021/acs.joc.8b02355 | JOC | 1 | 0 | 0 | |
| https://dx.doi.org/10.1021/acs.joc.8b02378 | JOC | 1 | 1 | 0 | 0 |
| https://dx.doi.org/10.1021/acs.joc.8b02389 | JOC | 1 | 0 | 0 | |
| https://dx.doi.org/10.1021/acs.joc.8b02575 | JOC | 1 | 1 | 0 | 0 |
| https://dx.doi.org/10.1021/acs.joc.8b02630 | JOC | 1 | 1 | 0 | 0 |
| https://dx.doi.org/10.1039/c7ob02040f | OBC | 1 | 0 | 0 | 0 |
| https://dx.doi.org/10.1039/c7ob02296d | OBC | 1 | 0 | 0 | |
| https://dx.doi.org/10.1039/c7ob02614e | OBC | 1 | 1 | 0 | 0 |
| https://dx.doi.org/10.1039/c7ob02677c | OBC | 1 | 0 | 0 | |
| https://dx.doi.org/10.1039/c7ob02769a | OBC | 1 | 1 | 0 | 0 |
| https://dx.doi.org/10.1039/c7ob02840g | OBC | 1 | 0 | 0 | |
| https://dx.doi.org/10.1039/c7ob03127k | OBC | 1 | 0 | 0 | |
| https://dx.doi.org/10.1039/c7ob03204h | OBC | 1 | 1 | 0 | 0 |
| https://dx.doi.org/10.1039/c8ob00005k | OBC | 1 | 0 | 0 | |
| https://dx.doi.org/10.1039/c8ob00056e | OBC | 1 | 0 | 0 | |
| https://dx.doi.org/10.1039/c8ob00078f | OBC | 1 | 0 | 0 | |
| https://dx.doi.org/10.1039/c8ob00099a | OBC | 1 | 1 | 0 | 0 |
| https://dx.doi.org/10.1039/c8ob00150b | OBC | 1 | 0 | 0 | |
| https://dx.doi.org/10.1039/c8ob00166a | OBC | 1 | 0 | 0 | |
| https://dx.doi.org/10.1039/c8ob00227d | OBC | 1 | 0 | 0 | |

| DOI | Journal | Synthesis | Pd used | Scavenger or chelator | Pd quant |
|---|---|---|---|---|---|
| https://dx.doi.org/10.1039/c8ob00323h | OBC | 1 | 1 | 0 | 0 |
| https://dx.doi.org/10.1039/C8OB00398J | OBC | 1 | 0 | 0 | |
| https://dx.doi.org/10.1039/c8ob00441b | OBC | 1 | 0 | 0 | |
| https://dx.doi.org/10.1039/c8ob00452h | OBC | 1 | 0 | 0 | |
| https://dx.doi.org/10.1039/c8ob00488a | OBC | 1 | 1 | 0 | 0 |
| https://dx.doi.org/10.1039/c8ob00500a | OBC | 1 | 0 | 0 | |
| https://dx.doi.org/10.1039/c8ob00630j | OBC | 1 | 0 | 0 | |
| https://dx.doi.org/10.1039/c8ob00670a | OBC | 1 | 0 | 0 | |
| https://dx.doi.org/10.1039/c8ob00706c | OBC | 1 | 0 | 0 | |
| https://dx.doi.org/10.1039/c8ob00840j | OBC | 1 | 0 | 0 | |
| https://dx.doi.org/10.1039/c8ob00877a | OBC | 1 | 0 | 0 | |
| https://dx.doi.org/10.1039/c8ob00946e | OBC | 1 | 0 | 0 | |
| https://dx.doi.org/10.1039/c8ob00979a | OBC | 1 | 0 | 0 | |
| https://dx.doi.org/10.1039/c8ob01002a | OBC | 1 | 1 | 0 | 0 |
| https://dx.doi.org/10.1039/c8ob01074a | OBC | 1 | 0 | 0 | |
| https://dx.doi.org/10.1039/c8ob01128a | OBC | 1 | 0 | 0 | |
| https://dx.doi.org/10.1039/c8ob01216d | OBC | 1 | 0 | 0 | |
| https://dx.doi.org/10.1039/c8ob01254g | OBC | 1 | 0 | 0 | |
| https://dx.doi.org/10.1039/c8ob01326h | OBC | 1 | 0 | 0 | |
| https://dx.doi.org/10.1039/c8ob01364k | OBC | 1 | 0 | 0 | |
| https://dx.doi.org/10.1039/c8ob01423j | OBC | 1 | 0 | 0 | |
| https://dx.doi.org/10.1039/c8ob01456f | OBC | 1 | 1 | 0 | 0 |
| https://dx.doi.org/10.1039/c8ob01460d | OBC | 1 | 0 | 0 | |
| https://dx.doi.org/10.1039/c8ob01504j | OBC | 1 | 0 | 0 | |
| https://dx.doi.org/10.1039/c8ob01507d | OBC | 1 | 0 | 0 | |
| https://dx.doi.org/10.1039/c8ob01640b | OBC | 1 | 0 | 0 | |
| https://dx.doi.org/10.1039/c8ob01701h | OBC | 1 | 0 | 0 | |
| https://dx.doi.org/10.1039/c8ob01702f | OBC | 1 | 1 | 0 | 0 |
| https://dx.doi.org/10.1039/c8ob01710g | OBC | 1 | 0 | 0 | |
| https://dx.doi.org/10.1039/c8ob01724g | OBC | 1 | 0 | 0 | |
| https://dx.doi.org/10.1039/c8ob01740a | OBC | 1 | 0 | 0 | |
| https://dx.doi.org/10.1039/c8ob01991f | OBC | 1 | 0 | 0 | |
| https://dx.doi.org/10.1039/c8ob02009d | OBC | 1 | 0 | 0 | |
| https://dx.doi.org/10.1039/c8ob02058b | OBC | 1 | 0 | 0 | |
| https://dx.doi.org/10.1039/c8ob02113a | OBC | 1 | 0 | 0 | |
| https://dx.doi.org/10.1039/c8ob02250j | OBC | 1 | 0 | 0 | |
| https://dx.doi.org/10.1039/c8ob02282h | OBC | 1 | 0 | 0 | |
| https://dx.doi.org/10.1039/c8ob02287a | OBC | 1 | 1 | 0 | 0 |
| https://dx.doi.org/10.1039/c8ob02304b | OBC | 1 | 0 | 0 | |
| https://dx.doi.org/10.1039/c8ob02432d | OBC | 1 | 0 | 0 | |

| DOI | Journal | Synthesis | Pd used | Scavenger or chelator | Pd quant |
|---|---|---|---|---|---|
| https://dx.doi.org/10.1039/c8ob02453g | OBC | 1 | 0 | 0 | |
| https://dx.doi.org/10.1039/c8ob02754d | OBC | 1 | 0 | 0 | |
| https://dx.doi.org/10.1039/c8ob02778a | OBC | 1 | 0 | 0 | |
| https://dx.doi.org/10.1039/c8ob02897d | OBC | 1 | 0 | 0 | |
| https://dx.doi.org/10.1039/c8ob90044b | OBC | 1 | 0 | 0 | |

Table S3A. Collated ICP-MS data showing average palladium levels (ppm) from three independent experiments

| Reaction | Crude, mean Pd ± SD (ppm) | Column, mean Pd ± SD (ppm) | Resin, mean Pd ± SD (ppm) |
|---|---|---|---|
| i (1, 3) | 677.0 ± 450.6 | 31.7 ± 54.8 | 14.3 ± 24.8 |
| ii (1, 3) | 1119.3 ± 1114.6 | 43.7 ± 48.0 | 0.0 |
| iii (1, 3) | 728.7 ± 467.0 | 57.3 ± 10.7 | 13.3 ± 23.1 |
| iv (1, 3) | 1980.0 ± 102.5 | 51.7 ± 30.6 | 31.3 ± 35.6 |
| i (1, 4) | 5136.0 ± 4424.2 | 1127.7 ± 1003.9 | 39.0 ± 33.9 |
| ii (1, 4) | 1454.7 ± 1761.0 | 58.7 ± 26.4 | 0.0 |
| iii (1, 4) | 1653.7 ± 2406.5 | 240.0 ± 357.7 | 10.3 ± 17.9 |
| iv (1, 4) | 951.7 ± 731.2 | 99.7 ± 77.6 | 9.0 ± 15.6 |
| iv (2, 5) | 2823.7 ± 4206.7 | 151.3 ± 213.1 | 0.0 |
| iv (6, 7) | 6208.3 ± 2188.3 | 663.7 ± 436.9 | 33.0 ± 35.8 |
| v (1, 8) | 4936.3 ± 2592.1 | 530.7 ± 420.3 | 16.0 ± 16.5 |
| vi (1, 9) | 3401.3 ± 644.3 | 165.3 ± 120.7 | 12.7 ± 21.9 |
| vii (10,11) | 207.0 ± 358.5 | 72.7 ± 43.8 | 34.7 ± 30.6 |

Table S3B. % Palladium removed from crude material following column chromatography, or following successive use of column chromatography and Si-TMT resin.

| Reaction | % Pd removed using column | % Pd removed using column & resin |
|---|---|---|
| i (1, 3) | 95.3 | 97.9 |
| ii (1, 3) | 96.1 | 100.0 |
| iii (1, 3) | 92.1 | 98.2 |
| iv (1, 3) | 97.4 | 98.4 |
| i (1, 4) | 78.0 | 99.2 |
| ii (1, 4) | 96.0 | 100.0 |
| iii (1, 4) | 85.5 | 99.4 |
| iv (1, 4) | 89.5 | 99.1 |
| iv (2, 5) | 94.6 | 100.0 |
| iv (6, 7) | 89.3 | 99.5 |
| v (1, 8) | 89.2 | 99.7 |
| vi (1, 9) | 95.1 | 99.6 |
| vii (10,11) | 64.9 | 83.3 |

Table S4A. Average residual palladium levels in reaction samples following Suzuki-Miyaura reaction (n=3, BLQ = below limit of quantification)

| Reaction | Crude, mean Pd ± SD (ppm) | Column, mean Pd ± SD (ppm) | Resin, mean Pd ± SD (ppm) |
|---|---|---|---|
| i (1, 3) | 677.0 ± 450.6 | 31.7 ± 54.8 | 14.3 ± 24.8 |
| ii (1, 3) | 1119.3 ± 1114.6 | 43.7 ± 48.0 | BLQ |
| iii (1, 3) | 728.7 ± 467.0 | 57.3 ± 10.7 | 13.3 ± 23.1 |
| iv (1, 3) | 1980.0 ± 102.5 | 51.7 ± 30.6 | 31.3 ± 35.6 |
| i (1, 4) | 5136.0 ± 4424.2 | 1127.7 ± 1003.9 | 39.0 ± 33.9 |
| ii (1, 4) | 1454.7 ± 1761.0 | 58.7 ± 26.4 | BLQ |
| iii (1, 4) | 1653.7 ± 2406.5 | 240.0 ± 357.7 | 10.3 ± 17.9 |
| iv (1, 4) | 951.7 ± 731.2 | 99.7 ± 77.6 | 9.0 ± 15.6 |
| iv (2, 5) | 2823.7 ± 4206.7 | 151.3 ± 213.1 | BLQ |
| iv (6, 7) | 6208.3 ± 2188.3 | 663.7 ± 436.9 | 33.0 ± 35.8 |

Table S4B. Average residual palladium levels in reaction samples following Buchwald-Hartwig reaction (n=3, BLQ = below limit of quantification)

| Reaction | Crude, mean Pd ± SD (ppm) | Column, mean Pd ± SD (ppm) | Resin, mean Pd ± SD (ppm) |
|---|---|---|---|
| (v) | 4936.3 ± 2592.1 | 530.7 ± 420.3 | 16.0 ± 16.5 |
| (vi) | 3401.3 ± 644.3 | 165.3 ± 120.7 | 12.7 ± 21.9 |

Table S4C. Average residual palladium levels in reaction samples following heterogenous metal on activated carbon reduction (n=3, BLQ = below limit of quantification)

| Reaction | Crude, mean Pd ± SD (ppm) | Column, mean Pd ± SD (ppm) | Resin, mean Pd ± SD (ppm) |
|---|---|---|---|
| (vii) | 207.0 ± 358.5 | 72.7 ± 43.8 | 34.7 ± 30.6 |

Table S5A. Residual palladium levels in crude reaction samples following aqueous work-up only (BLQ = below limit of quantification)

| Reaction | Run 1 Sample mass (mg) | Run 1 Pd (ng/mg) | Run 2 Sample mass (mg) | Run 2 Pd (ng/mg) | Run 3 Sample mass (mg) | Run 3 Pd (ng/mg) | Mean Pd ± SD (ppm) |
|---|---|---|---|---|---|---|---|
| (i)-1 | 16.2 | 496 | 19.9 | 345 | 21.4 | 1190 | 677.0 ± 450.6 |
| (ii)-1 | 29.2 | 353 | 19.6 | 2398 | 35.7 | 607 | 1119.3 ± 1114.6 |
| (iii)-1 | 20 | 1267 | 18 | 486 | 25.2 | 433 | 728.7 ± 467.0 |
| (iv)-1 | 20 | 1884 | 21 | 2088 | 21.5 | 1968 | 1980.0 ± 102.5 |
| (i)-2 | 21.2 | 5190 | 20 | 9533 | 16.5 | 685 | 5136.0 ± 4424.2 |
| (ii)-2 | 20 | 552 | 24.6 | 3484 | 20.6 | 328 | 1454.7 ± 1761.0 |
| (iii)-2 | 22.8 | 478 | 20.2 | 61 | 20 | 4422 | 1653.7 ± 2406.5 |
| (iv)-2 | 15.5 | 1474 | 20 | 1265 | 19.1 | 116 | 951.7 ± 731.2 |
| (iv)-3 | 20.7 | 7681 | 17 | 358 | 29.2 | 432 | 2823.7 ± 4206.7 |
| (iv)-4 | 22.9 | 3743 | 20 | 7921 | 20 | 6961 | 6208.3 ± 2188.3 |
| (v) | 21.1 | 7617 | 18 | 4749 | 19.4 | 2443 | 4936.3 ± 2592.1 |
| (vi) | 21.9 | 3931 | 16.8 | 3589 | 20.5 | 2684 | 3401.3 ± 644.3 |
| (vii) | 20.3 | BLQ | 20.9 | 621 | 20 | BLQ | 207.0 ± 358.5 |

Table S5B. Residual palladium levels in reaction samples following automated (Biotage™) column chromatography (BLQ = below limit of quantification)

| Reaction | Run 1 Sample mass (mg) | Run 1 Pd (ng/mg) | Run 2 Sample mass (mg) | Run 2 Pd (ng/mg) | Run 3 Sample mass (mg) | Run 3 Pd (ng/mg) | Mean Pd ± SD (ppm) |
|---|---|---|---|---|---|---|---|
| (i)-1 | 19.4 | BLQ | 22.8 | BLQ | 20.8 | 95 | 31.7 ± 54.8 |
| (ii)-1 | 20.8 | 95 | 21.2 | 36.0 | 27.3 | BLQ | 43.7 ± 48.0 |
| (iii)-1 | 20 | 63 | 19 | 64 | 13.5 | 45 | 57.3 ± 10.7 |
| (iv)-1 | 20 | 35 | 24.4 | 33 | 17 | 87 | 51.7 ± 30.6 |
| (i)-2 | 20.8 | 1924 | 20 | 1459 | 15 | BLQ | 1127.7 ± 1003.9 |
| (ii)-2 | 20 | 30 | 23.2 | 82 | 11 | 64.0 | 58.7 ± 26.4 |
| (iii)-2 | 21.5 | 41 | 21 | 26 | 20 | 653 | 240.0 ± 357.7 |
| (iv)-2 | 21 | 182 | 20 | 89 | 19.3 | 28 | 99.7 ± 77.6 |
| (iv)-3 | 21.8 | 395 | 13 | BLQ | 19.6 | 59 | 151.3 ± 213.1 |
| (iv)-4 | 12.1 | 186 | 20 | 1043 | 20 | 762 | 663.7 ± 436.9 |
| (v) | 23.9 | 1015 | 18 | 315 | 26.6 | 262 | 530.7 ± 420.3 |
| (vi) | 21.9 | 238 | 25.9 | 232 | 25.4 | 26 | 165.3 ± 120.7 |
| (vii) | 21.4 | 79 | 22 | 26 | 17 | 113 | 72.7 ± 43.8 |

Table S5C Residual palladium levels in reaction samples following automated (Biotage™) column chromatography and metal scavenging using Si-TMT resin (BLQ = below limit of quantification, n/a = not applicable)

| | Run 1 | | Run 2 | | Run 3 | | |
|---|---|---|---|---|---|---|---|
| Reaction | Sample mass (mg) | Pd (ng/mg) | Sample mass (mg) | Pd (ng/mg) | Sample mass (mg) | Pd (ng/mg) | Mean Pd ± SD (ppm) |
| (i)-1 | 21.1 | 43 | 20.9 | BLQ | 20.3 | BLQ | 14.3 ± 24.8 |
| (ii)-1 | 33.9 | BLQ | 25.1 | BLQ | 20.9 | BLQ | BLQ |
| (iii)-1 | 20 | BLQ | 21 | 40 | 23.4 | BLQ | 13.3 ± 23.1 |
| (iv)-1 | 21.3 | 70 | 25.1 | BLQ | 24 | 24 | 31.3 ± 35.6 |
| (i)-2 | 22.6 | 56 | 18.1 | 61 | 20 | BLQ | 39.0 ± 33.9 |
| (ii)-2 | 20 | BLQ | 20.2 | BLQ | 10 | BLQ | BLQ |
| (iii)-2 | 20.5 | BLQ | 26.5 | BLQ | 20 | 31 | 10.3 ± 17.9 |
| (iv)-2 | 23 | BLQ | 21.2 | 27 | 21.9 | BLQ | 9.0 ± 15.6 |
| (iv)-3 | 20.3 | BLQ | 20 | BLQ | 18.3 | BLQ | BLQ |
| (iv)-4 | 19.5 | 28 | 20.6 | 71 | 20 | BLQ | 33.0 ± 35.8 |
| (v) | 70.2 | 15 | 17 | 33 | 28.8 | BLQ | 16.0 ± 16.5 |
| (vi) | 20.6 | BLQ | 24.4 | BLQ | 21.3 | 38 | 12.7 ± 21.9 |
| (vii) | 20.2 | 46 | 20.6 | BLQ | 13 | 58 | 34.7 ± 30.6 |

Table S6 Additional observations made during the pilot study concerning experimental variability

| |
|---|
| Use of different palladium sources for catalytic reactions seemed to affect the levels of residual palladium. This preliminary study was not designed to explore this factor, but warrants further investigation. |
| In some cases where a 'conventional' workup was used (aqueous wash / liquid-liquid extraction, drying agent, filtration and chromatographic purification), we noted residual levels of several hundred ppm of palladium in many of the isolated products. If this process was carried out less efficiently (e.g. with an inexperienced operator, use of short silica 'plugs') or when reaction 'telescoping' products, significantly higher levels of metal impurity may be carried through into isolated reaction products. |
| Significant operator-based variability in residual palladium levels was found, even when using a standardised experimental procedure (*data not shown*). |
| Variations in the work-up procedure were investigated; these were found to profoundly influence the levels of residual palladium (*data not shown*) |
| A range of methods for scavenging residual palladium metal are available, and may prove equally or more effective when compared to the single Si-TMT resin-based technique employed here. |

## General Experimental

Analytical and quality standards were upheld as for typical organic synthesis. NMR and LCMS analysis was used as the primary method of structure confirmation, and to ensure overall purity levels would be suitable (>95% purity) for submission to a typical *in vitro* biological screening assay following column chromatography. Some of the compounds prepared in this study were known and so provided for a large body of existing characterisation data, which was used to confirm authenticity of the reaction products. Others were entirely novel and are being reported for the first time. Sources of comparative data and CAS numbers (where relevant) for all materials are provided below, along with representative analytical data obtained during the study.

All reactions involving moisture-sensitive reagents were carried out under a nitrogen or argon atmosphere using standard vacuum line techniques and glassware that was flame dried and cooled under nitrogen before use. Water was purified by an Elix® UV-10 system. All other reagents were used as supplied (analytical or HPLC grade) without prior purification. Thin layer chromatography was performed on aluminium plates coated with 60 F254 silica. Flash column chromatography was performed on a Biotage SP4 automated flash column chromatography platform. Melting points were recorded on a EZ-Melt Automated Melting Point Apparatus (EZ Melt) and are uncorrected. NMR spectra were recorded on Bruker Avance III spectrometers (at 400 or 500 MHz) using the deuterated solvent stated and at RT. The field was locked by external referencing to the relevant deuteron resonance. Accurate mass measurements were run on either a Bruker MicroTOF internally calibrated with polyalanine, or a Micromass GCT instrument fitted with a Scientific Glass Instruments BPX5 column (15 m $\times$ 0.25 mm) using amyl acetate as a lock mass. Column chromatography was performed on a Biotage Isolera One 3.0 with the default settings for a KP-Sil 50 g cartridge and monitored at 254 and 280 nm. For residual palladium analysis CEMAS procedure CAM- 0338/001 was followed. Therein sample digestion took place using super pure hydrochloric and super pure nitric acid in a Mars6 Microwave Digester. The residue was quantitatively transferred to a volumetric flask and made to volume with super pure water. ICP-MS analysis was undertaken using an Agilent G8421A 7800. Data analysis software was Agilent ICPMS MassHunter with ECM XT with secure data storage. The limit of detection for palladium was 10 ng / mL.

Palladium loading in the different reaction classes were 5 mol% (wrt halide starting material) for Suzuki-Miyaura reactions, 1 mol% for Buchwald-Hartwig conditions (both homogeneous catalysis), and 10 mol% for heterogeneous nitro group reductions.

## Abbreviations

app = apparent; BLQ = below limit of quantification; CAS = Chemical Abstracts Service; DME = dimethoxymethane; ESI = electrospray ionisation; HRMS = high resolution mass spectrometry; ICP-MS = Inductively coupled plasma mass spectrometry; LCMS = liquid chromatography-mass spectrometry; LiHMDS = lithium hexamethyldisilazide; NMR = nuclear magnetic resonance; RT = room (ambient) temperature; Si-TMT = 2,4,6-trimercaptotriazine silica gel

## Work-Up Details

ICP-MS analysis was performed on all samples at three different points in the work-up / purification process to determine levels of residual palladium.

1. ***'Crude' sample:*** Each reaction was evaporated directly, or subjected to a conventional aqueous work-up, and repeated organic extraction. This was intended to be a reference for 'worst case' metal contamination. In some cases, the standard work-up was preceded by filtration of the complete reaction mixture through a plug of Celite™. The combined organic phases were washed successively with saturated ammonium chloride solution, water and brine before being dried over anhydrous magnesium sulfate and concentrated under reduced pressure using a rotary evaporator.
2. ***'Column' sample:*** The extent to which trace palladium impurities are removed from crude material using column chromatography, and which would likely be NMR and LCMS silent is unclear. Since material of this nature would commonly be tested in biological assays, we wanted to establish how effective routine column chromatography is in removing residual palladium, and therefore whether additional purification steps would be required. To remove potential variability which would result from manual flash chromatography, the crude mixture was loaded onto an automated column chromatography system for purification and purified using the default parameters.
3. ***'Resin' sample:*** The post-column residue was suspended in solvent and treated with 0.35 weight equivalents of Si-TMT resin (used as it is cheap, readily available, and can be purchased on scale). Importantly, some non-peer reviewed data were available verifying its effectiveness at removing palladium. The resulting suspension was stirred gently for one hour at room temperature before being filtered, dried, and evaporated.

**General Procedure 1: Suzuki-Miyaura reaction**

Aryl halide (1 mmol), boronate species (1.2 mmol), $K_2CO_3$ (2 mmol), catalyst (0.05 mmol) and ligand (where applicable, see Figure 1 for specified amount) were added to a 5 mL microwave vial and purged with argon for 5 min. Degassed DME (2 mL) and $H_2O$ (1 mL) were added, the vial purged for a further 1 min, and the reaction was heated to 80 °C for 5 h. The reaction mixture was allowed to cool to RT, passed through Celite™ and the solvents were evaporated *in vacuo* (crude sample). Purification with flash column chromatography (10 – 50% EtOAc in pentane for 5-(4-fluorophenyl)-1*H*-indole, 30 – 50% EtOAc in pentane for 5-(1-methyl-1*H*-pyrazol-4-yl)-1*H*-indole, 30 – 50% EtOAc in pentane for 1-methyl-5-(1-methyl-1*H*-pyrazol-4-yl)-1*H*-indole and 6-(1-methyl-1*H*-pyrazol-4-yl)quinazoline) afforded the desired products.

**General Procedure 2: Buchwald-Hartwig**

Aryl halide (1 mmol), amine (1.2 mmol), catalyst (0.01 mmol) and ligand (0.04 mmol) were added to a 5 mL microwave vial and purged with argon for 5 min. LiHMDS (2.2 mmol, 1 M in THF) was added, and the reaction was stirred at 65 °C for 24 h. The reaction mixture was allowed to cool to RT, quenched with $NH_4Cl$, followed by successive extractions using EtOAc (2 x 10 mL), dried over anhydrous $MgSO_4$ and the solvents were evaporated *in vacuo*. Trituration with $Et_2O$ or $CH_2Cl_2$ (crude sample) was followed by purification using flash column chromatography (20% EtOAc in pentane or 30 – 40% EtOAc in pentane as eluent for *N*-(4-fluorophenyl)-1*H*-indol-5-amine and 4-(1*H*-indol-5-yl)morpholine, respectively) afforded the desired products.

**Pd catalysed nitro group reduction, 1-methyl-1*H*-indol-5-amine (11)**

1-Methyl-5-nitro-1*H*-indole (**10**) (1 mmol) in EtOH (3 mL) was degassed thoroughly with nitrogen. Pd/C (10%, 0.1 mmol) was added, and degassed with hydrogen. The reaction was stirred under a hydrogen

atmosphere at RT for 24 h. The reaction mixture was passed through Celite™ and the solvents were evaporated *in vacuo* (crude sample). Purification using flash column chromatography (30 – 50% EtOAc in pentane as eluent) afforded the title compound.

**Resin use**

ISOLUTE Si-TMT from Biotage was used. Scavenging capacity 0.3 mmol / g.

**Procedure:** the flash chromatography purified product was dissolved in $CH_2Cl_2$, and Si-TMT (350 mg resin/ 1 g compound) was added. The mixture was stirred at room temperature for 1 h, filtered and concentrated *in vacuo* to give the desired product.

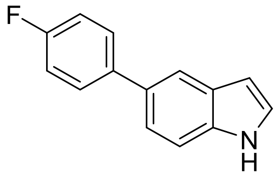


**5-(4-Fluorophenyl)-1*H*-indole (3) [CAS No 144104-44-9]**

*Comparative analytical data from:*

https://doi.org/10.1016/j.tetlet.2019.151040

https://doi.org/10.1039/C7CC06267B

https://doi.org/10.1002/ejoc.201403214

https://doi.org/10.1021/jo047975c

mp 93.4 – 94.4 °C {lit. 90 – 92 °C; 90 – 93 °C}; $^1$H NMR (400 MHz, $CDCl_3$) *δ* 8.19 (br s, 1H), 7.80 (dt, *J* = 1.7, 0.8 Hz, 1H), 7.62 – 7.55 (m, 2H), 7.46 (dt, *J* = 8.4, 0.9 Hz, 1H), 7.39 (dd, *J* = 8.4, 1.8 Hz, 1H), 7.29 – 7.23 (m, 1H), 7.17 – 7.07 (m, 2H), 6.61 (ddd, *J* = 3.1, 2.1, 1.0 Hz, 1H); $^{13}$C NMR (101 MHz, $CDCl_3$) *δ* 162.1 (d, *J* = 244.9 Hz), 138.8 (d, *J* = 3.3 Hz), 135.4, 132.6, 128.9 (d, *J* = 7.9 Hz), 128.5, 125.1, 121.9, 119.3, 115.5 (d, *J* = 21.3 Hz), 111.4, 103.1; $^{19}$F NMR (376 MHz, $CDCl_3$) *δ* -116.7 – -118.1 (m). The $^1$H and $^{13}$C NMR data are in accordance to spectra previously reported in the literature; HRMS (ESI): *m/z* calculated for $C_{14}H_{10}FN$ + H [M + H]$^+$: 212.08700; Found: 212.08708.

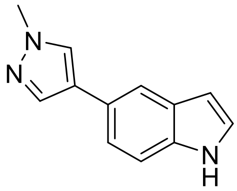


**5-(1-Methyl-1*H*-pyrazol-4-yl)-1*H*-indole (4)**

mp 158.0 – 158.4 °C; $^1$H NMR (400 MHz, $CDCl_3$) *δ* 8.17 (s, 1H), 7.78 (d, *J* = 1.0 Hz, 1H), 7.76 – 7.72 (m, 1H), 7.60 (d, *J* = 0.7 Hz, 1H), 7.39 (dt, *J* = 8.6, 1.1 Hz, 1H), 7.32 (dd, *J* = 8.3, 1.7 Hz, 1H), 7.21 (dd, *J* = 3.2, 2.4 Hz, 1H), 6.55 (ddd, *J* = 3.2, 2.1, 1.0 Hz, 1H), 3.95 (s, 3H); $^{13}$C NMR (101 MHz, $CDCl_3$) *δ* 136.9, 135.0, 128.6, 126.7, 124.9, 124.7, 124.6, 120.8, 117.6, 111.5, 102.8, 39.2; HRMS (ESI): *m/z* calculated for $C_{12}H_{11}N_3$ + H [M + H]$^+$: 198.1026; Found: 198.1026.

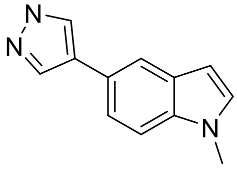


**1-Methyl-5-(1-methyl-1*H*-pyrazol-4-yl)-1*H*-indole (5)**

mp 191.5 – 192.3 °C; $^1$H NMR (400 MHz, $CDCl_3$) *δ* 7.78 (d, *J* = 0.8 Hz, 1H), 7.72 (dd, *J* = 1.7, 0.8 Hz, 1H), 7.60 (d, *J* = 0.8 Hz, 1H), 7.35 (dd, *J* = 8.5, 1.6 Hz, 1H), 7.31 (dt, *J* = 8.5, 0.8 Hz, 1H), 7.05 (d, *J* = 3.1 Hz, 1H), 6.47 (dd, *J* = 3.1, 0.8 Hz, 1H), 3.95 (s, 3H), 3.80 (s, 3H); $^{13}$C NMR (101 MHz, $CDCl_3$) δ 136.9, 135.9, 129.5, 129.1, 126.6, 124.7, 124.2, 120.3, 117.7, 109.6, 101.0, 39.1, 33.0; HRMS (ESI): *m/z* calculated for $C_{13}H_{13}N_3$ + H [M + H]$^+$: 212.1182; Found: 212.1182.

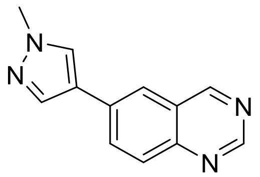

**6-(1-Methyl-1*H*-pyrazol-4-yl)quinazoline (6)**

mp 154.8 – 155.2 °C; $^{1}$H NMR (400 MHz, $CDCl_3$) *δ* 9.37 (s, 1H), 9.27 (s, 1H), 8.04 – 8.03 (m, 2H), 7.95 (t, *J* = 1.3 Hz, 1H), 7.90 (d, *J* = 0.9 Hz, 1H), 7.77 (d, *J* = 0.8 Hz, 1H), 3.99 (s, 3H); $^{13}$C NMR (101 MHz, $CDCl_3$) *δ* 160.0, 154.9, 149.1, 137.2, 132.8, 132.7, 129.1, 127.7, 125.8, 122.0, 121.8, 39.4; HRMS (ESI): *m/z* calculated for $C_{12}H_{10}N_4$ + H $[M + H]^+$: 211.0978; Found: 211.0979.

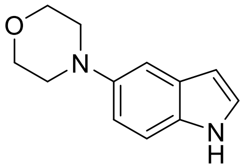

**4-(1*H*-Indol-5-yl)morpholine (8) [CAS No 245117-16-2]**

*Comparative analytical data from:*

*https://doi.org/10.1021/acs.joc.7b00201*

*https://doi.org/10.1021/ol0514754*

mp 127.5 – 128.8 °C {lit. 134 – 135 °C; 132 – 135 °C}; $^{1}$H NMR (400 MHz, $CDCl_3$) *δ* 8.08 (s, 1H), 7.31 (d, *J* = 8.7 Hz, 1H), 7.21 – 7.12 (m, 2H), 6.97 (dd, *J* = 8.8, 2.4 Hz, 1H), 6.48 (t, *J* = 2.7 Hz, 1H), 4.01 – 3.82 (m, 4H), 3.14 (dd, *J* = 5.7, 3.6 Hz, 4H); $^{13}$C NMR (101 MHz, $CDCl_3$) δ 146.1, 131.7, 128.6, 124.8, 115.4, 111.7, 107.6, 102.6, 67.4, 52.2; The $^{1}$H and $^{13}$C NMR data are in accordance to spectra previously reported in the literature; HRMS (ESI): *m/z* calculated for $C_{12}H_{14}ON_2$ + H $[M + H]^+$: 203.11789; Found: 203.11792.

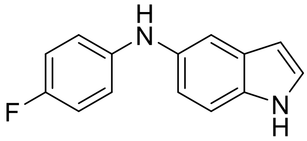

***N*-(4-Fluorophenyl)-1*H*-indol-5-amine (9)**

mp 112.4 – 113.9 °C; $^{1}$H NMR (400 MHz, $CDCl_3$) δ 8.08 (s, 1H), 7.36 (d, *J* = 2.1 Hz, 1H), 7.33 (d, *J* = 8.5 Hz, 1H), 7.20 (t, *J* = 2.8 Hz, 1H), 6.98 (dd, *J* = 8.6, 2.1 Hz, 1H), 6.91 (app td, *J* = 5.7, 3.3 Hz, 4H), 6.48 (t, *J* = 2.4 Hz, 1H), 5.48 (s, 1H); $^{13}$C NMR (101 MHz, $CDCl_3$) δ 157.0 (d, *J* = 237.2 Hz), 142.4, 136.1, 132.6, 128.8, 125.1, 117.7, 117.3 (d, *J* = 7.5 Hz), 115.8 (d, *J* = 22.4 Hz), 112.2, 111.9, 102.5; $^{19}$F NMR (376 MHz, $CDCl_3$) *δ* -125.3 (ddd, *J* = 13.1, 8.0, 5.0 Hz); HRMS (ESI): *m/z* calculated for $C_{14}H_{11}FN_2$ + H $[M + H]^+$: 227.0979; Found: 227.0979.

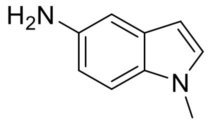

**1-Methyl-1*H*-indol-5-amine (11) [CAS No 102308-97-4]**

*Comparative analytical data from:*

*https://doi.org/10.1080/14756366.2020.1800666*

*https://doi.org/10.1021/acs.orglett.9b04400*

*https://doi.org/10.1080/00397919808005084*

mp 102.6 – 102.8 °C {lit. 103 – 103.9 °C; 105 °C}; $^{1}$H NMR (400 MHz, $CDCl_3$) $\delta$ 7.13 (d, *J* = 8.6 Hz, 1H), 6.95 (app dd, *J* = 12.3, 2.9 Hz, 2H), 6.70 (dd, *J* = 8.6, 2.1 Hz, 1H), 6.29 (d, *J* = 3.8 Hz, 1H), 3.73 (s, 3H); $^{13}$C NMR (101 MHz, $CDCl_3$) $\delta$ 139.3, 132.0, 129.4, 129.2, 112.6, 109.8, 105.8, 99.6, 32.9; The $^{1}$H and $^{13}$C NMR data are in accordance to spectra previously reported in the literature; HRMS (ESI): *m/z* calculated for $C_9H_{10}N_2$ + H $[M + H]^+$: 147.09167; Found: 147.09155.

**Spectra**

## 5-(4-Fluorophenyl)-1*H*-indole (3) [CAS No 144104-44-9]

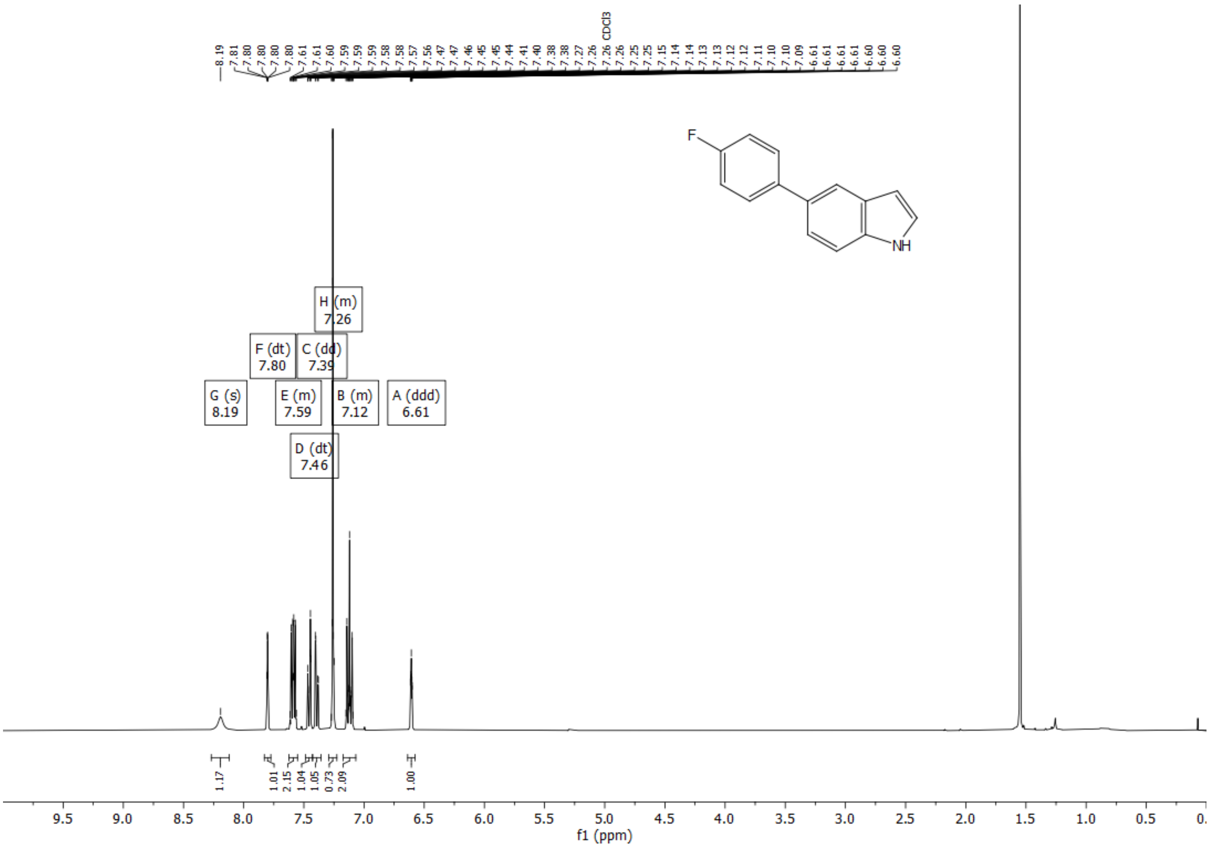

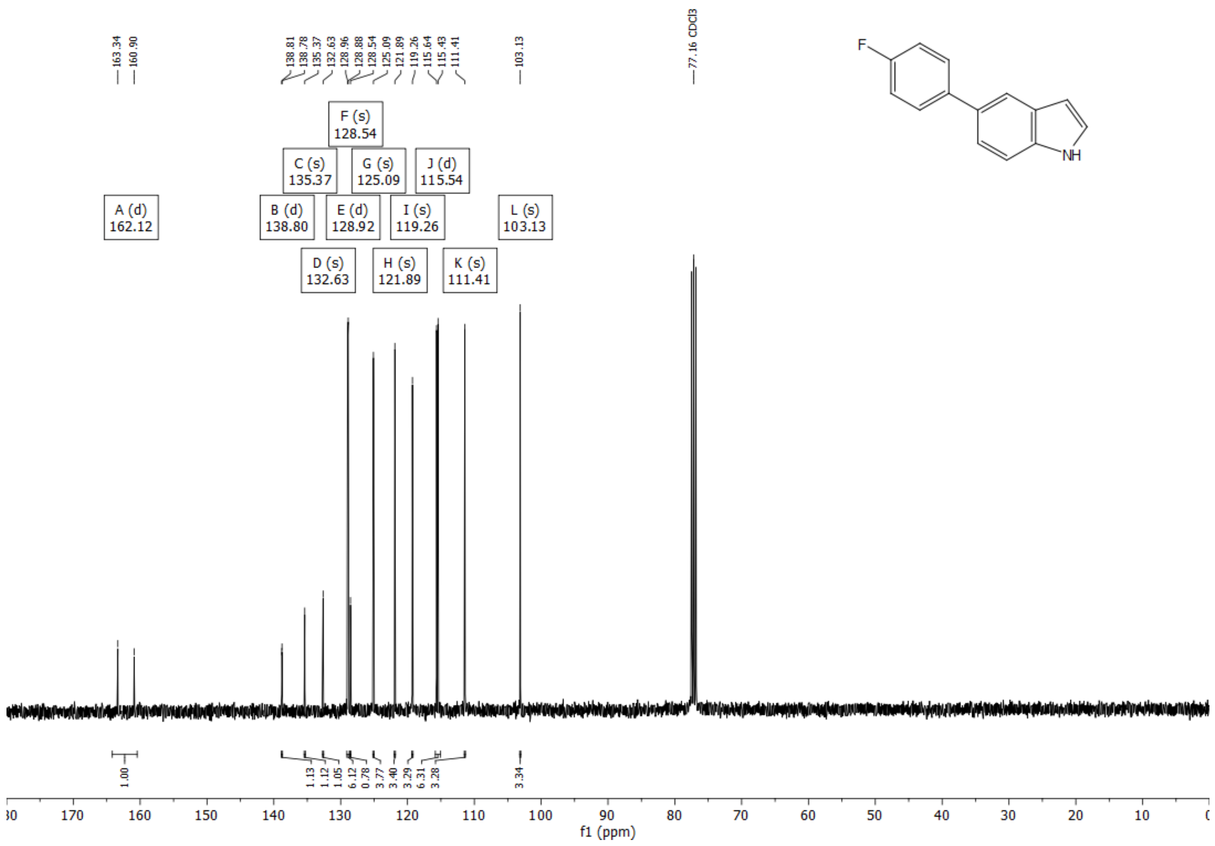
163.34
160.90
138.81
138.78
135.37
132.63
128.96
128.88
128.54
125.09
121.89
119.26
115.64
115.43
111.41
103.13
77.16 CDCl3
F
NH
A (d)
162.12
B (d)
138.80
C (s)
135.37
D (s)
132.63
E (d)
128.92
F (s)
128.54
G (s)
125.09
H (s)
121.89
I (s)
119.26
J (d)
115.54
K (s)
111.41
L (s)
103.13
f1 (ppm)

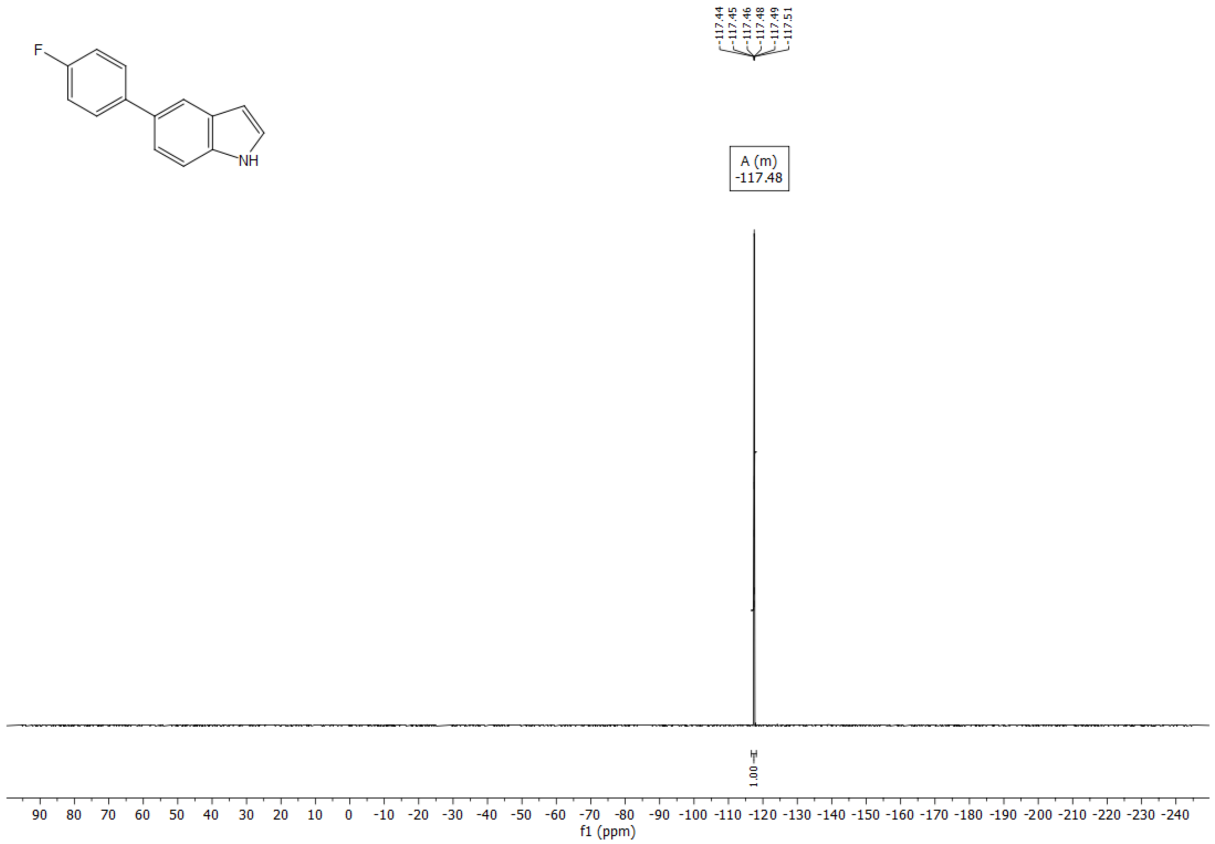
F
NH
-117.44
-117.45
-117.46
-117.48
-117.49
-117.51
A (m)
-117.48
1.00
f1 (ppm)

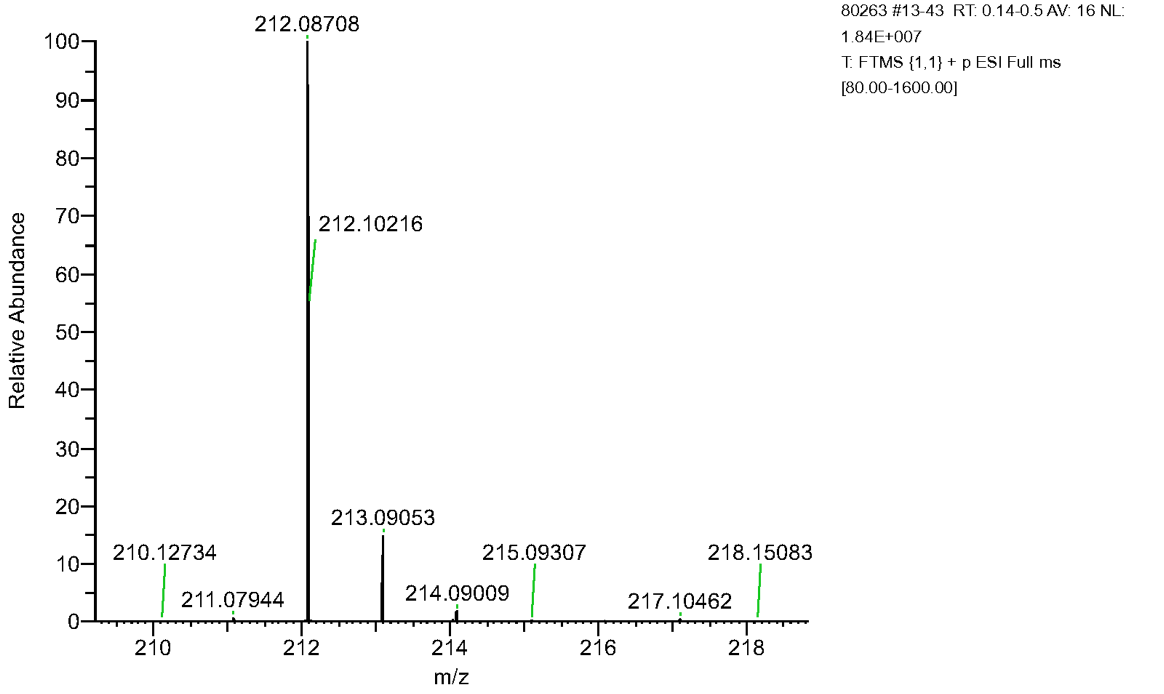


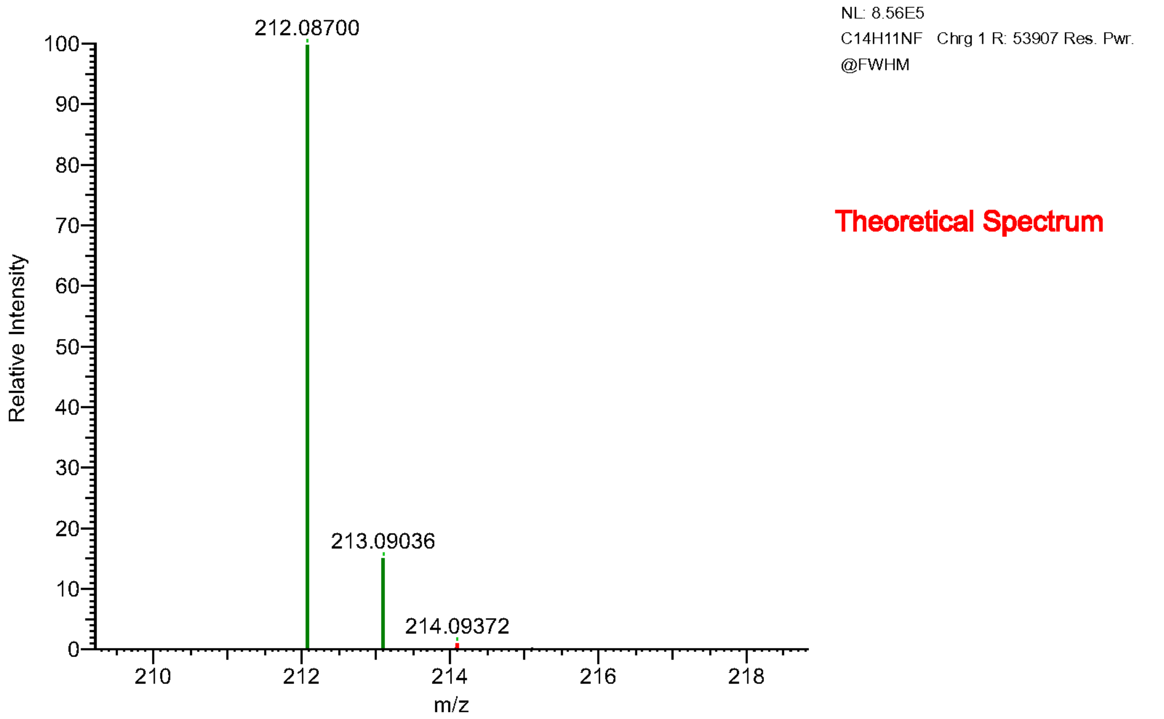


| Peak Mass | Display Formula | Delta [ppm] | Theo. mass | Rank | MSMS Matched Frag... |
|---|---|---|---|---|---|
| 212.08708 | $C_{14}H_{11}NF$ | 0.35 | 212.08700 | 1 | (Collection) |

## 5-(1-Methyl-1*H*-pyrazol-4-yl)-1*H*-indole (4)

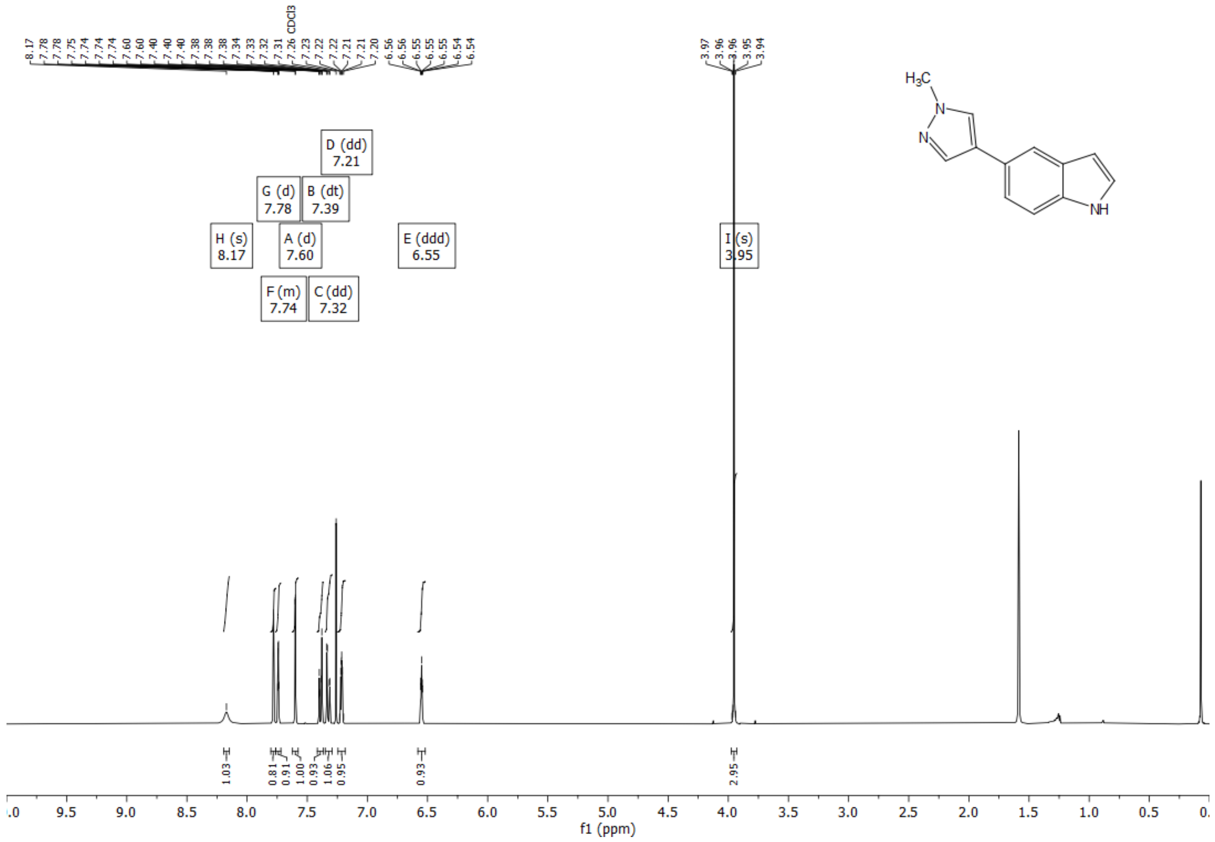


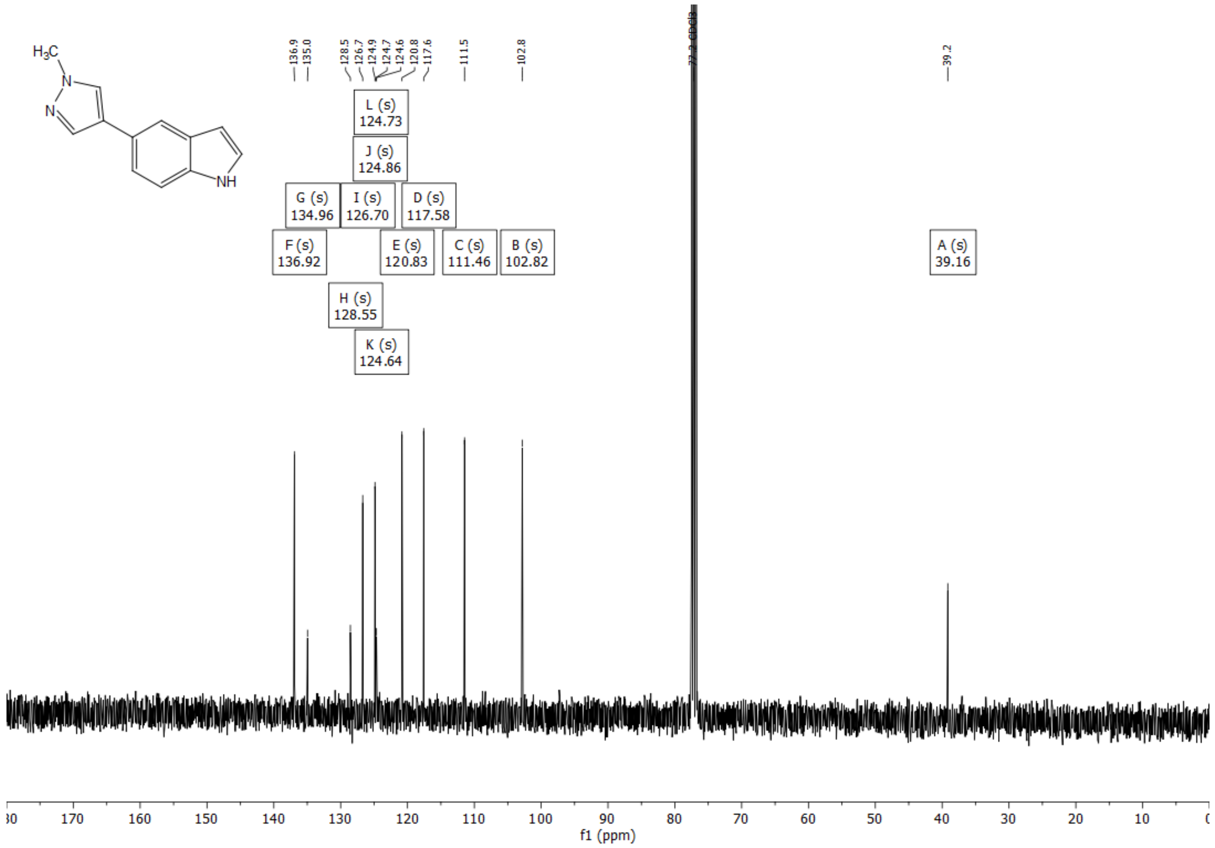

Z:\data\2021\(03) Mar\ESI86947.raw 26/03/2021 6:07 pm

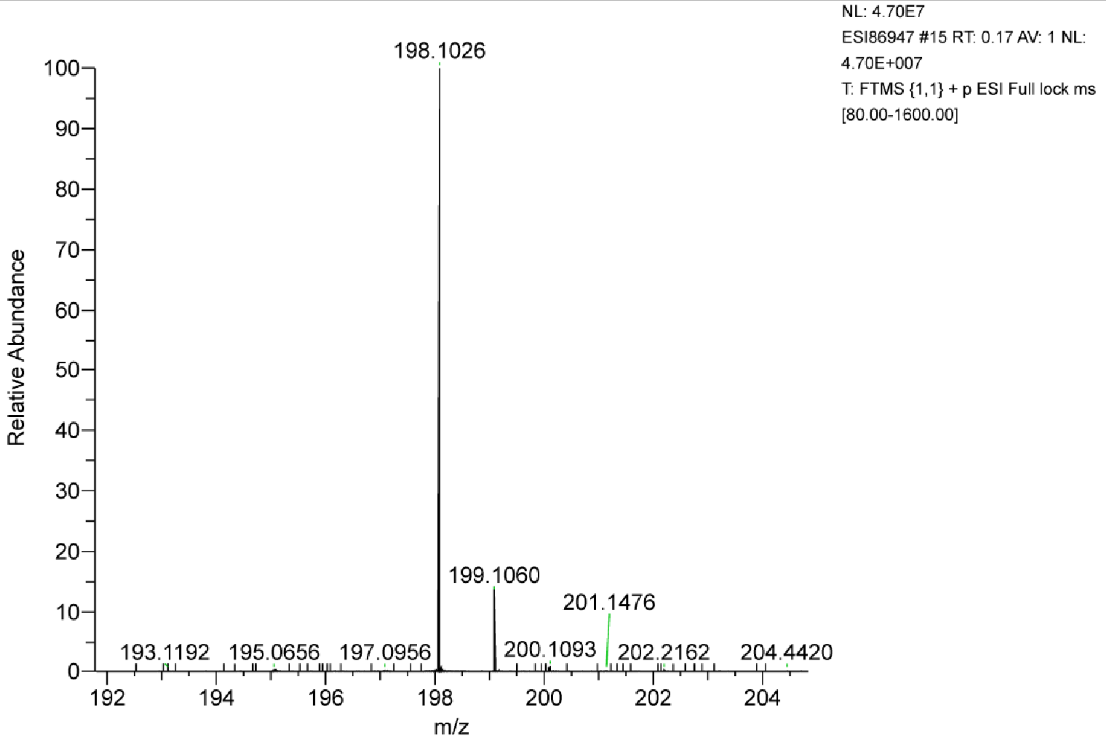


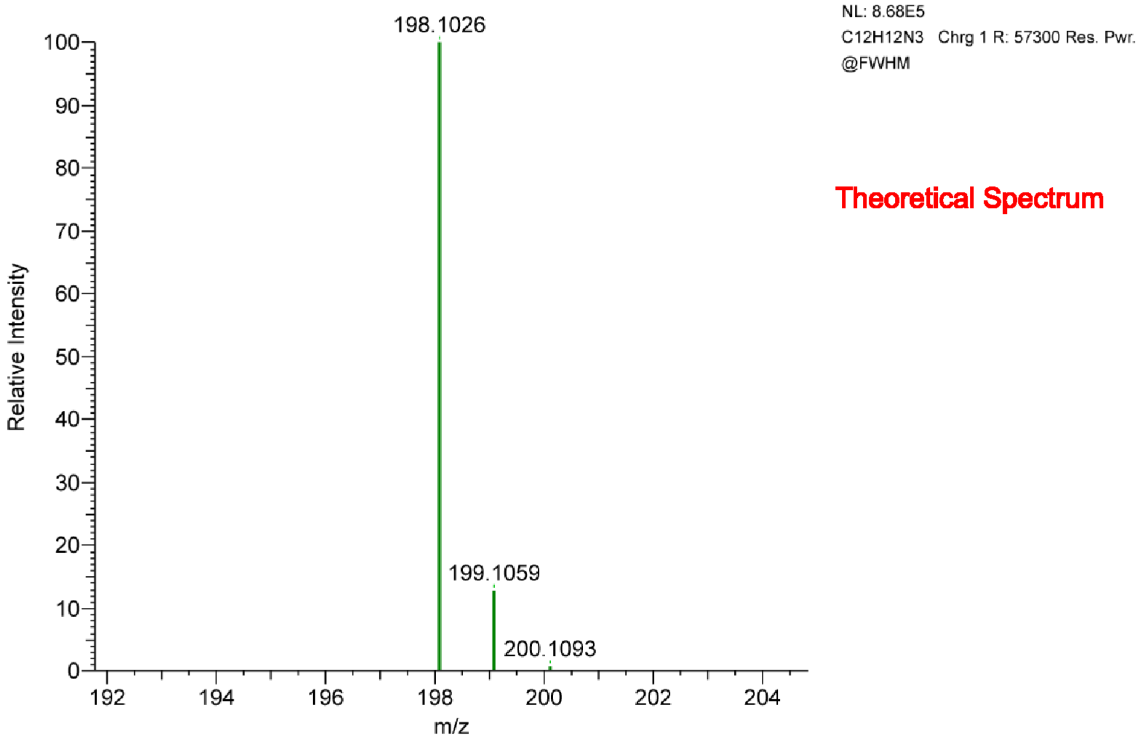


| Peak Mass | Display... | Combin... | RDB | Delta [p... | Theo. m... | Rank | Combin... | # Match... | # Misse... | MS Cov.... | Pattern... | MSMS... |
|---|---|---|---|---|---|---|---|---|---|---|---|---|
| 198.1026 | C[illegible]H[illegible]N[illegible] | 70.471 | 8.50 | 0.05 | 198.10 | 1 | 97.575 | 5 | 0 | 99.081 | 100 | (Collec |

**1-Methyl-5-(1-methyl-1*H*-pyrazol-4-yl)-1*H*-indole (5)**

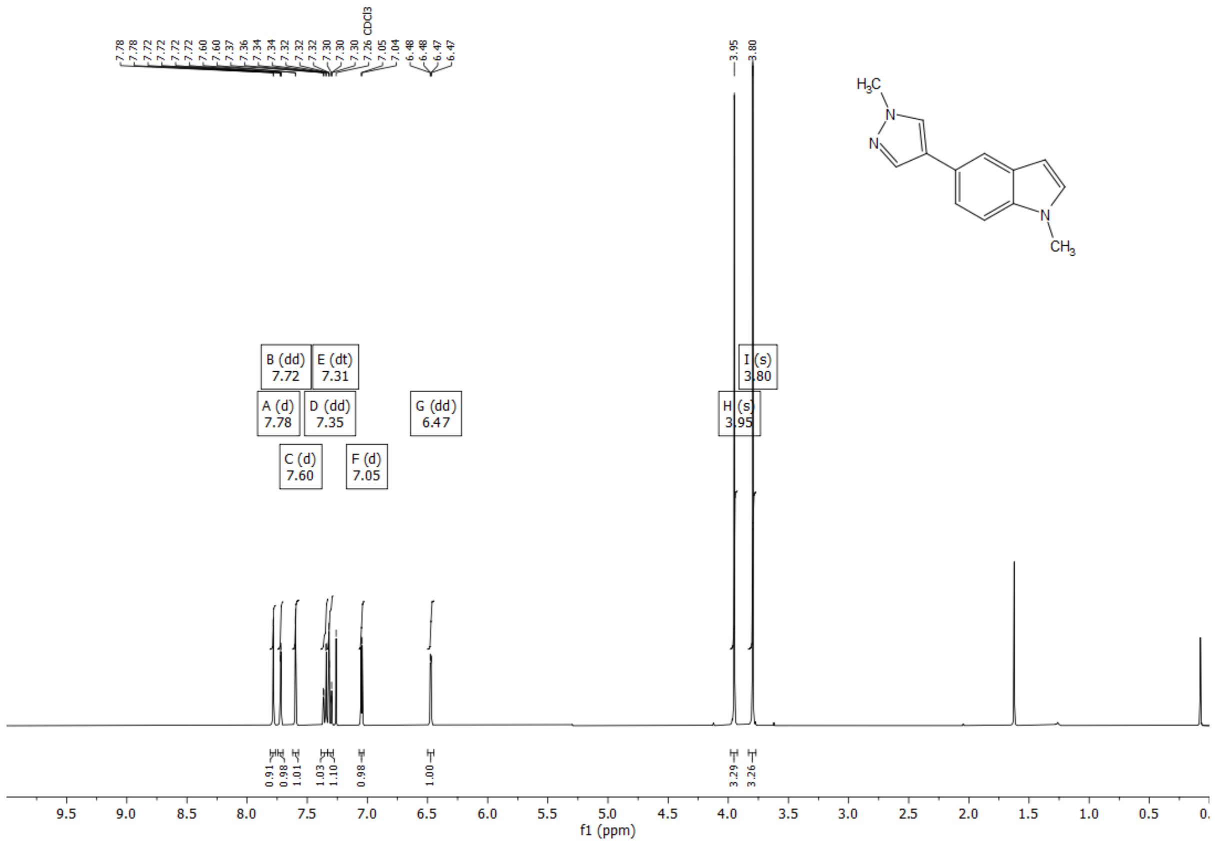

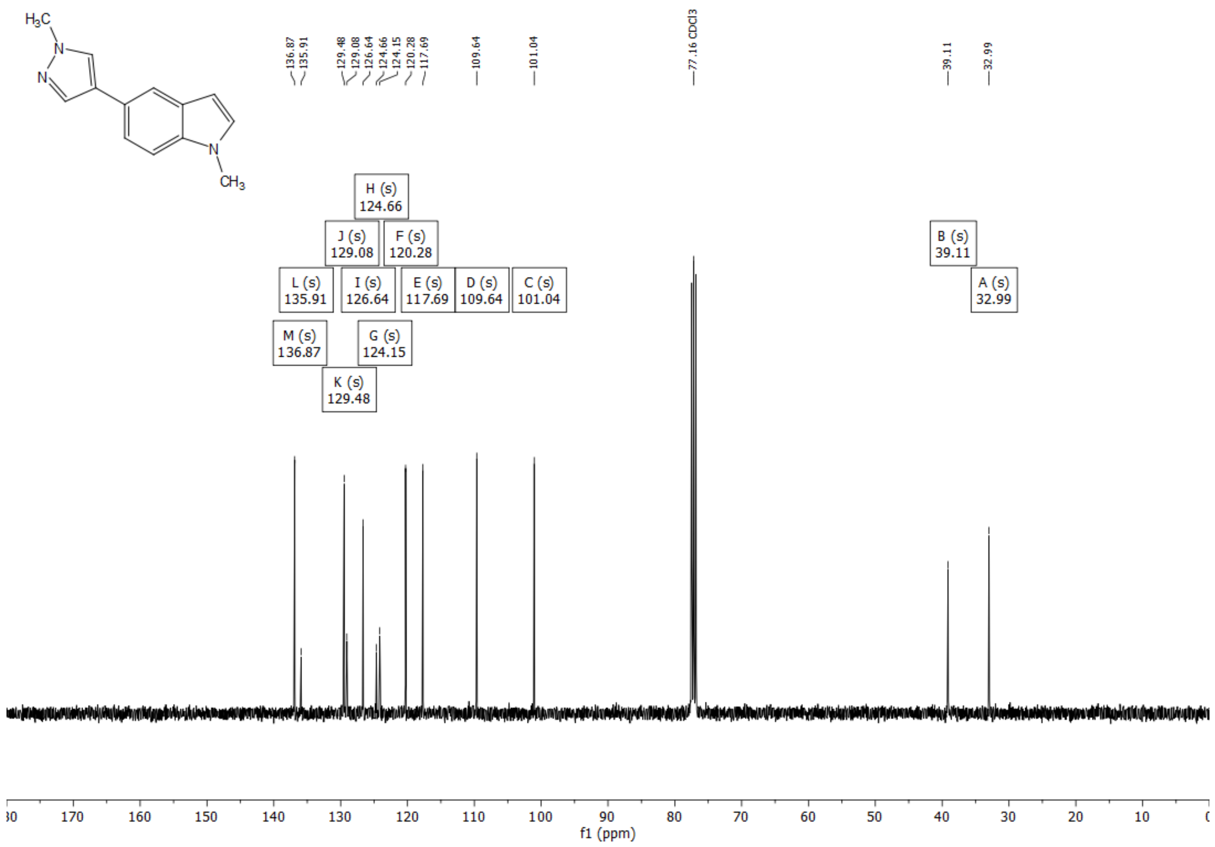

Z:\data\2021\(03) Mar\ESI86946.raw 26/03/2021 6:06 pm

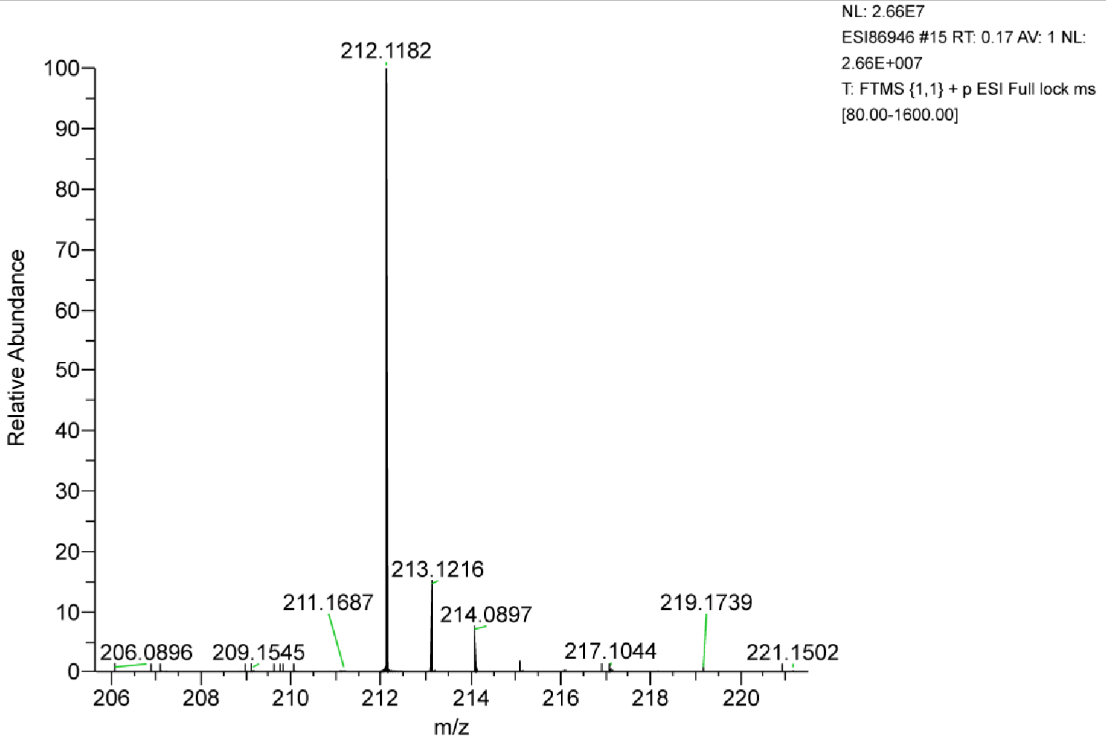


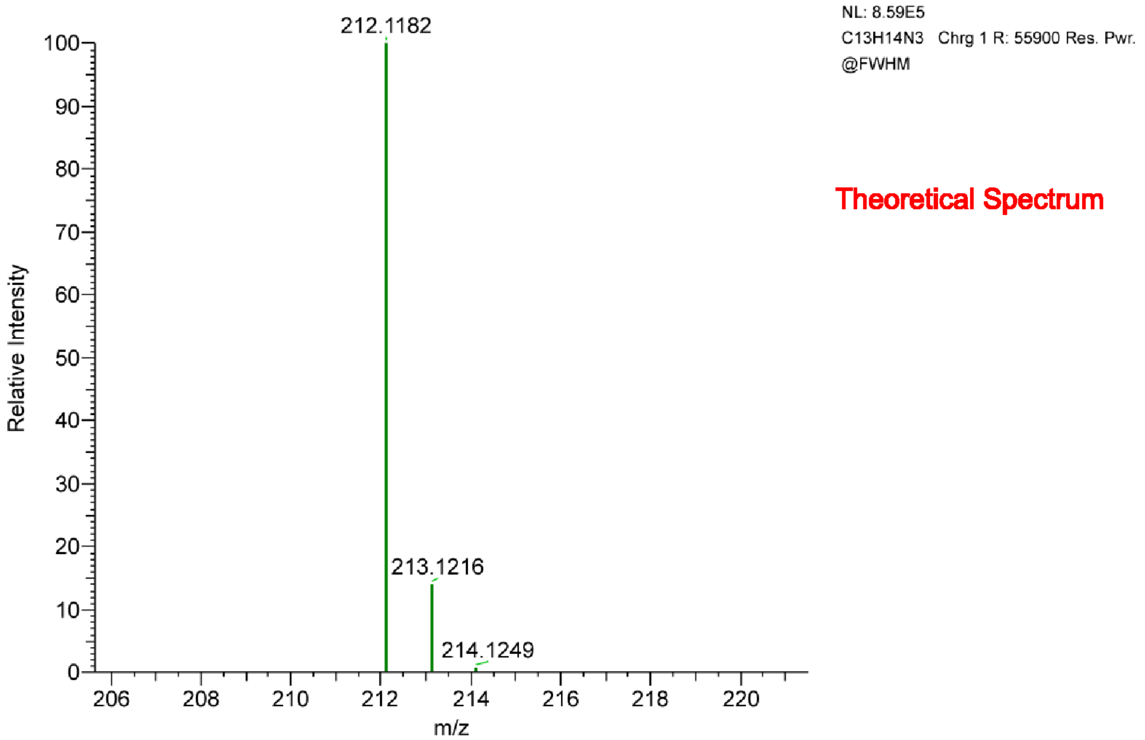


| Peak Mass | Display... | Combin... | RDB | Delta [p... | Theo. m... | Rank | Combin... | # Match... | # Misse... | MS Cov.... | Pattern... | MSMS... |
|---|---|---|---|---|---|---|---|---|---|---|---|---|
| 212.1182 | [illegible] | 61.049 | 8.50 | 0.08 | 212.11 | 1 | 92.956 | 5 | 0 | 94.729 | 100 | (Collec |

**6-(1-Methyl-1*H*-pyrazol-4-yl)quinazoline (7)**

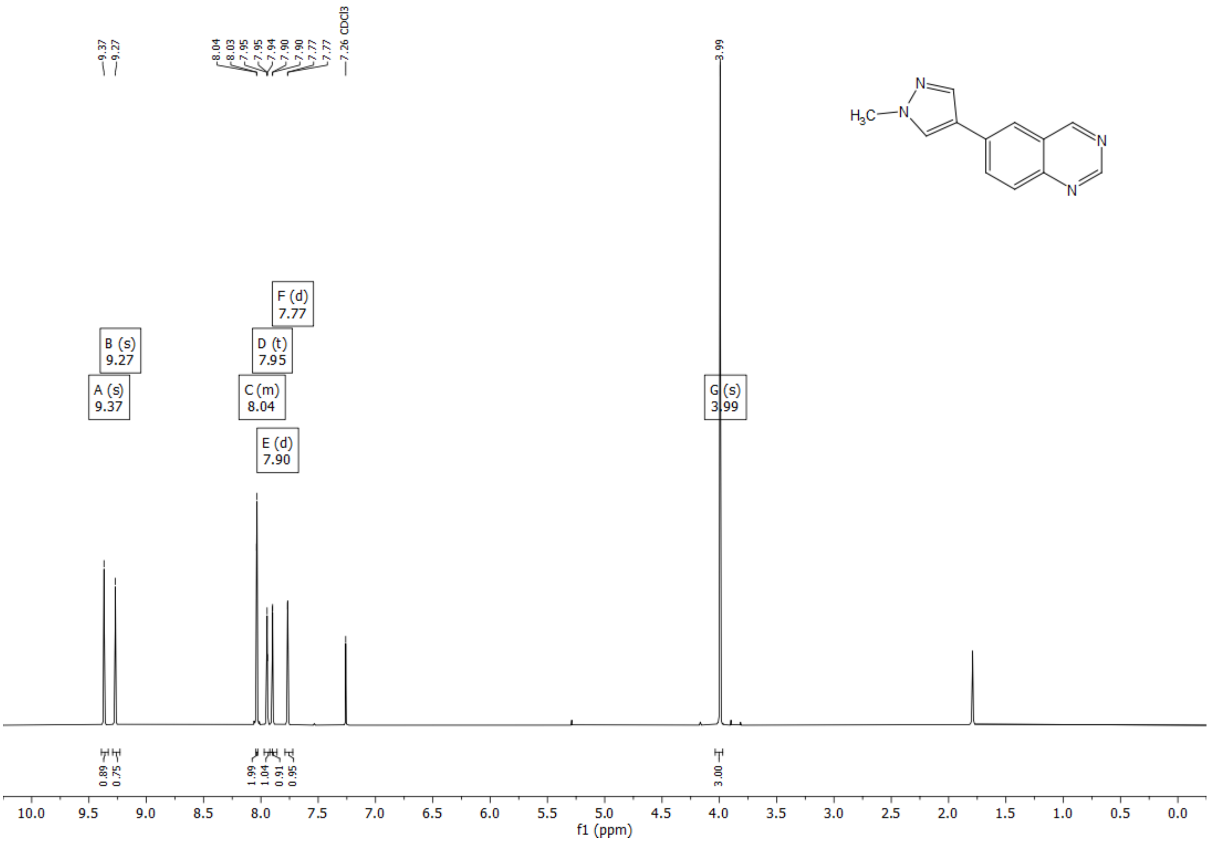


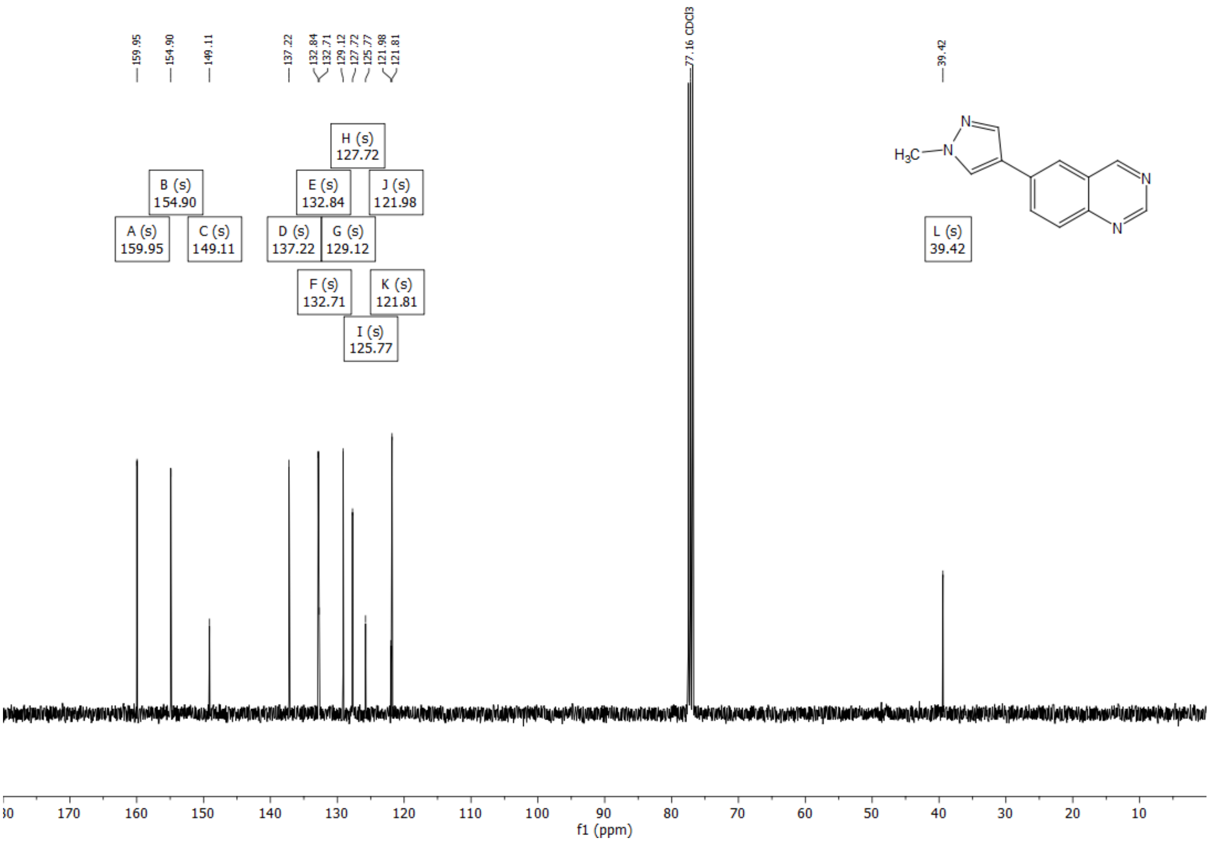

NL: 1.14E8
ESI80068 #15 RT: 0.17 AV: 1 NL: 1.14E+008
T: FTMS {1,1} + p ESI Full lock ms [80.00-1600.00]

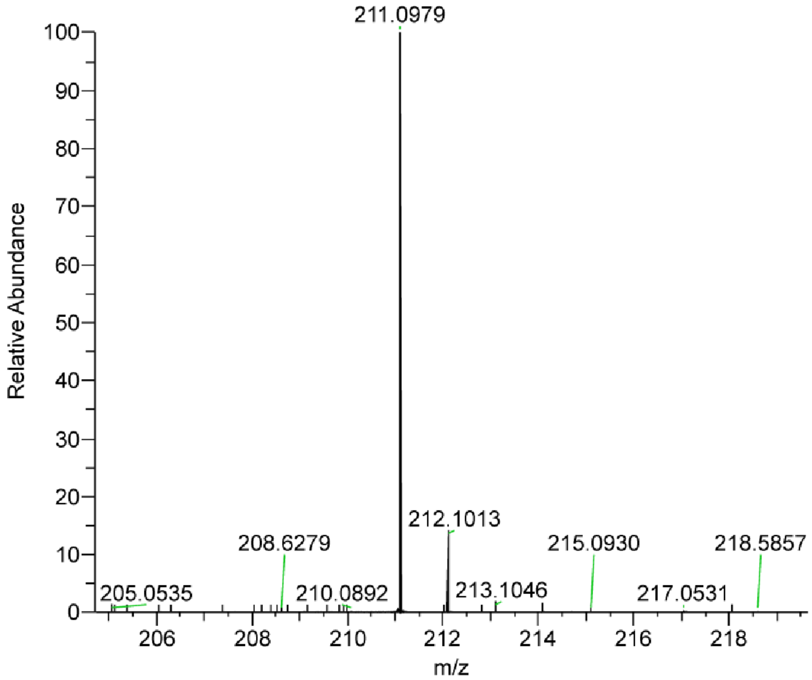


NL: 8.65E5
C12H11N4 Chrg 1 R: 56000 Res. Pwr. @FWHM

**Theoretical Spectrum**

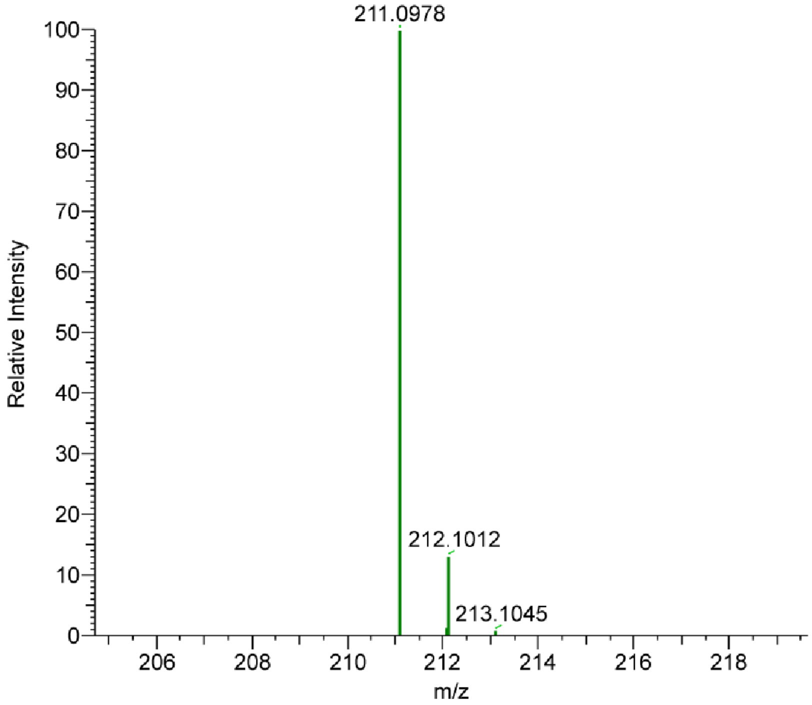


| Peak Mass | Display... | Combin... | RDB | Delta [p... | Theo. m... | Rank | Combin... | # Match... | # Misse... | MS Cov.... | Pattern... | MSMS... |
|---|---|---|---|---|---|---|---|---|---|---|---|---|
| 211.0979 | $C_{12}H_{11}N_4$ | 53.385... | 9.50 | 0.37 | 211.09... | 1 | 97.507... | 5 | 0 | 99.959... | 100 | (Collec... |

## 4-(1*H*-Indol-5-yl)morpholine (8)

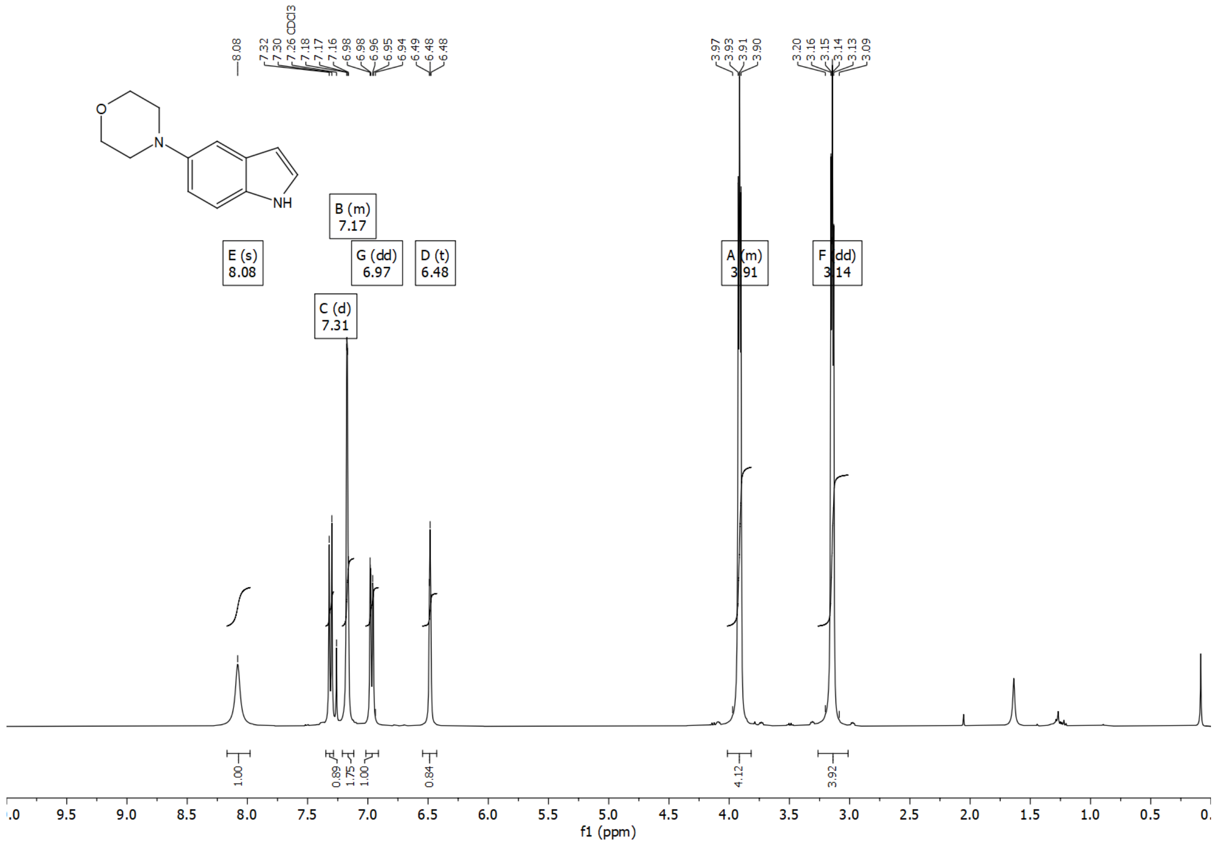


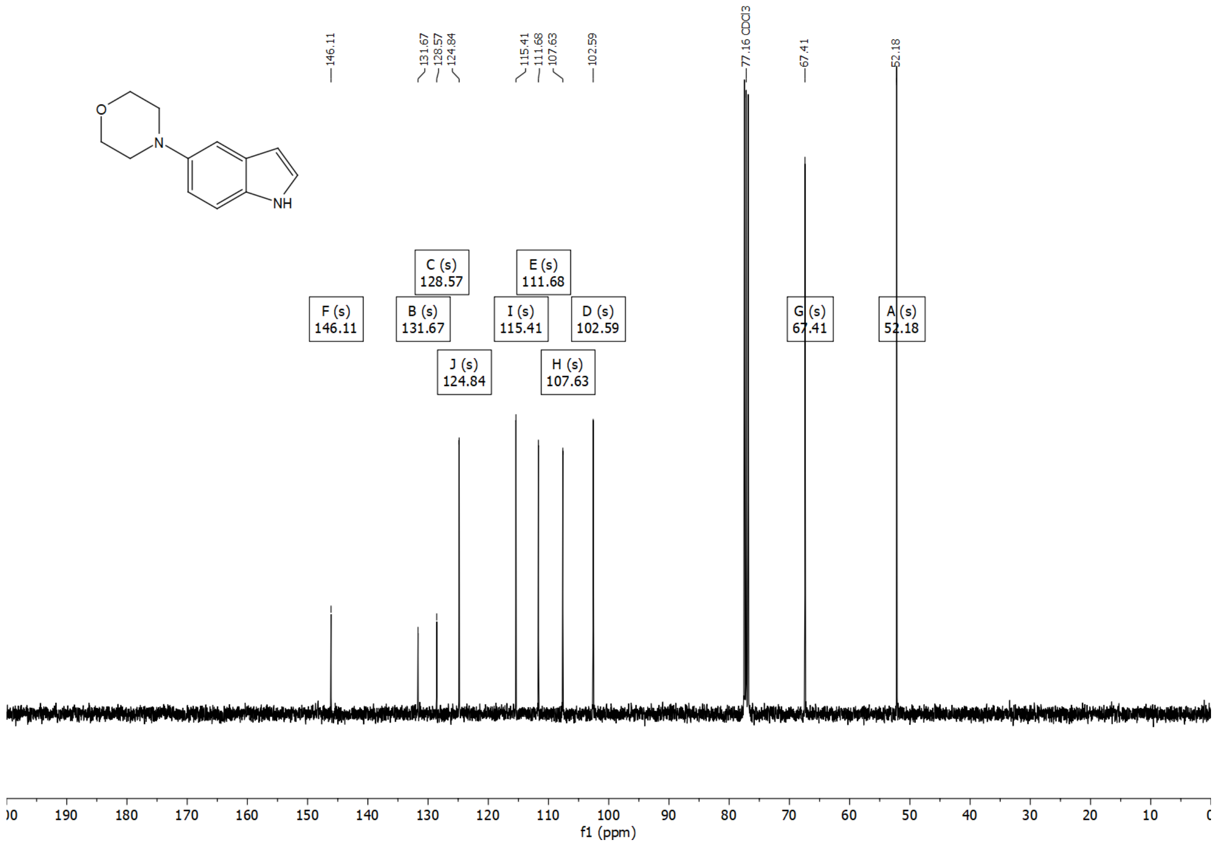

Z:\data\Sep19\ESI79647.raw 16/09/2019 10:58 am

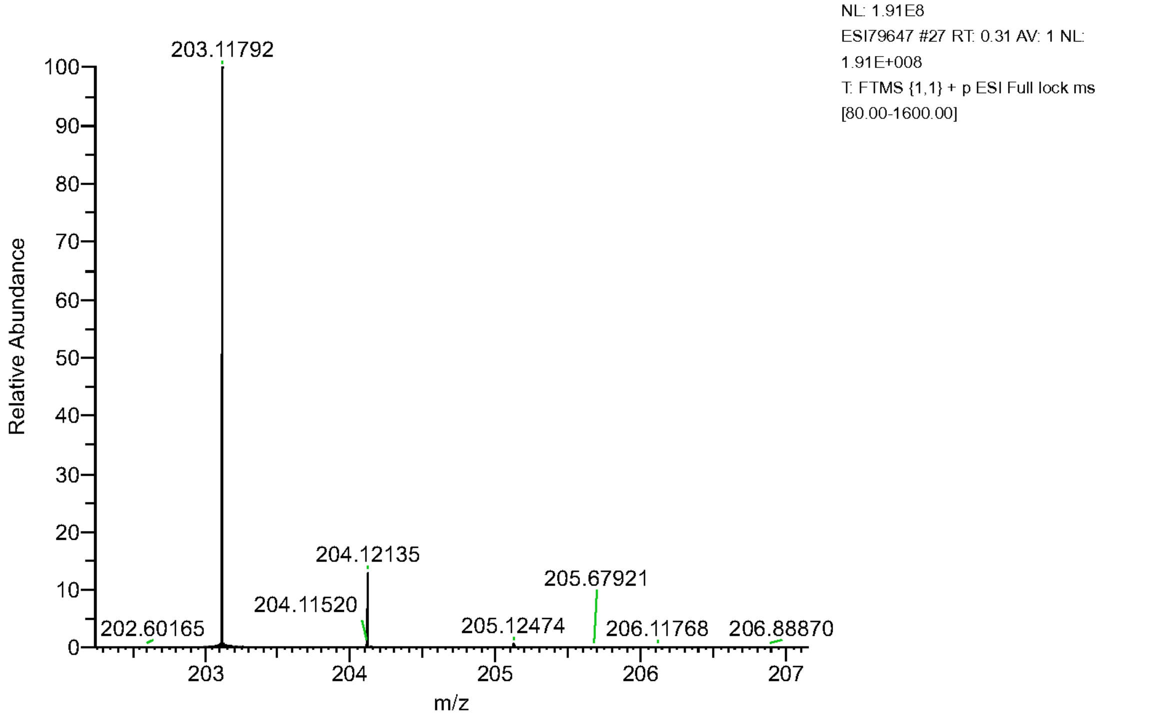


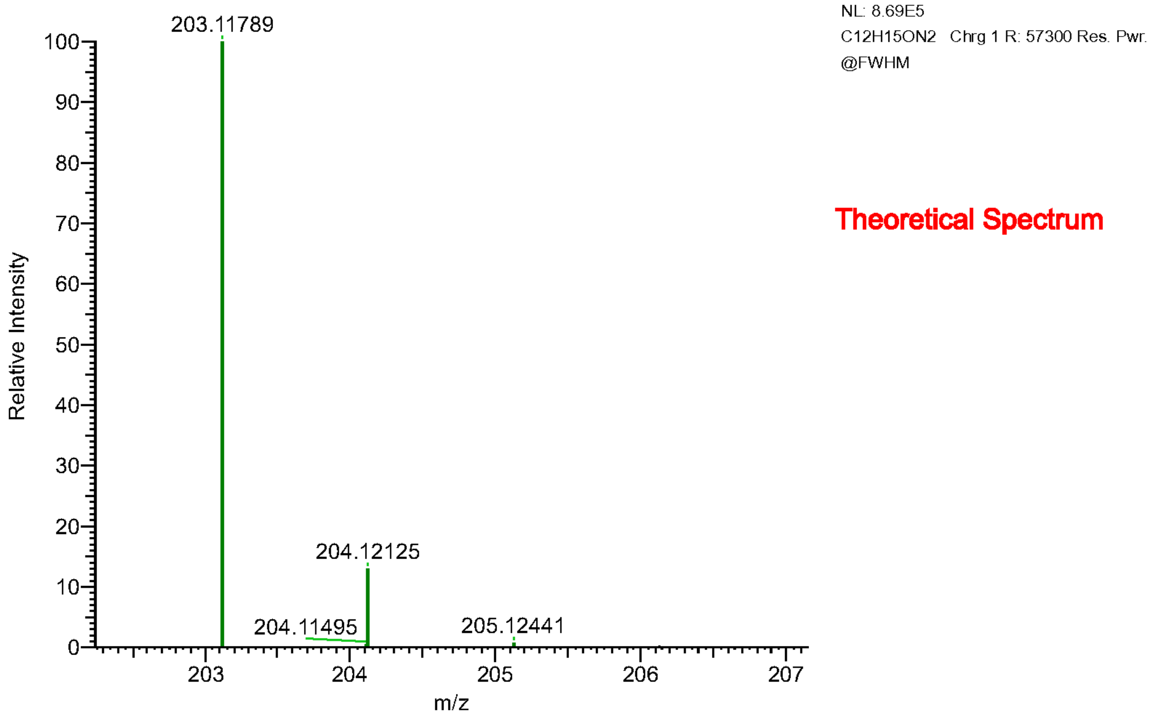


| Peak Mass | Display Formula | Delta [ppm] | Theo. mass | Rank | MSMS Matched Frag... |
|---|---|---|---|---|---|
| 203.11792 | $C_{12}H_{15}ON_2$ | 0.15 | 203.11789 | 1 | (Collection) |

## N-(4-Fluorophenyl)-1*H*-indol-5-amine (9)

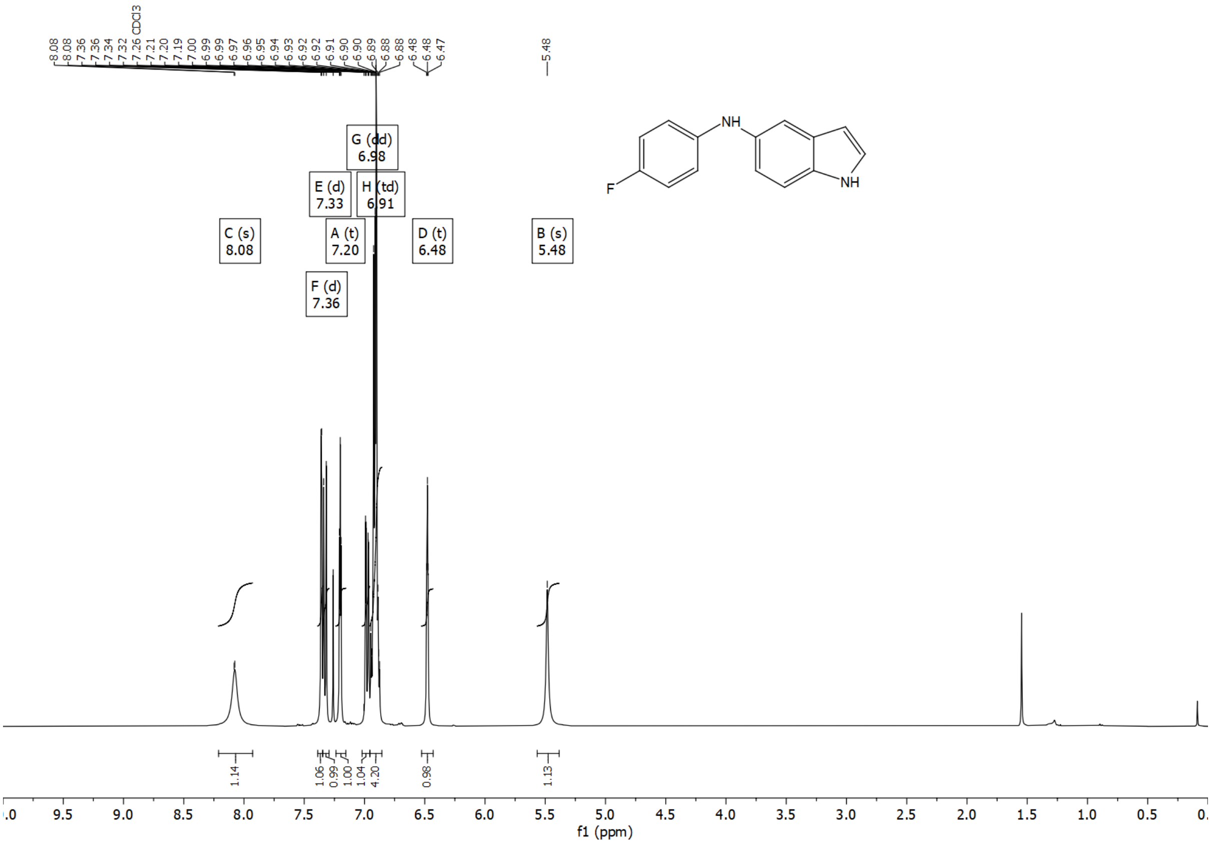


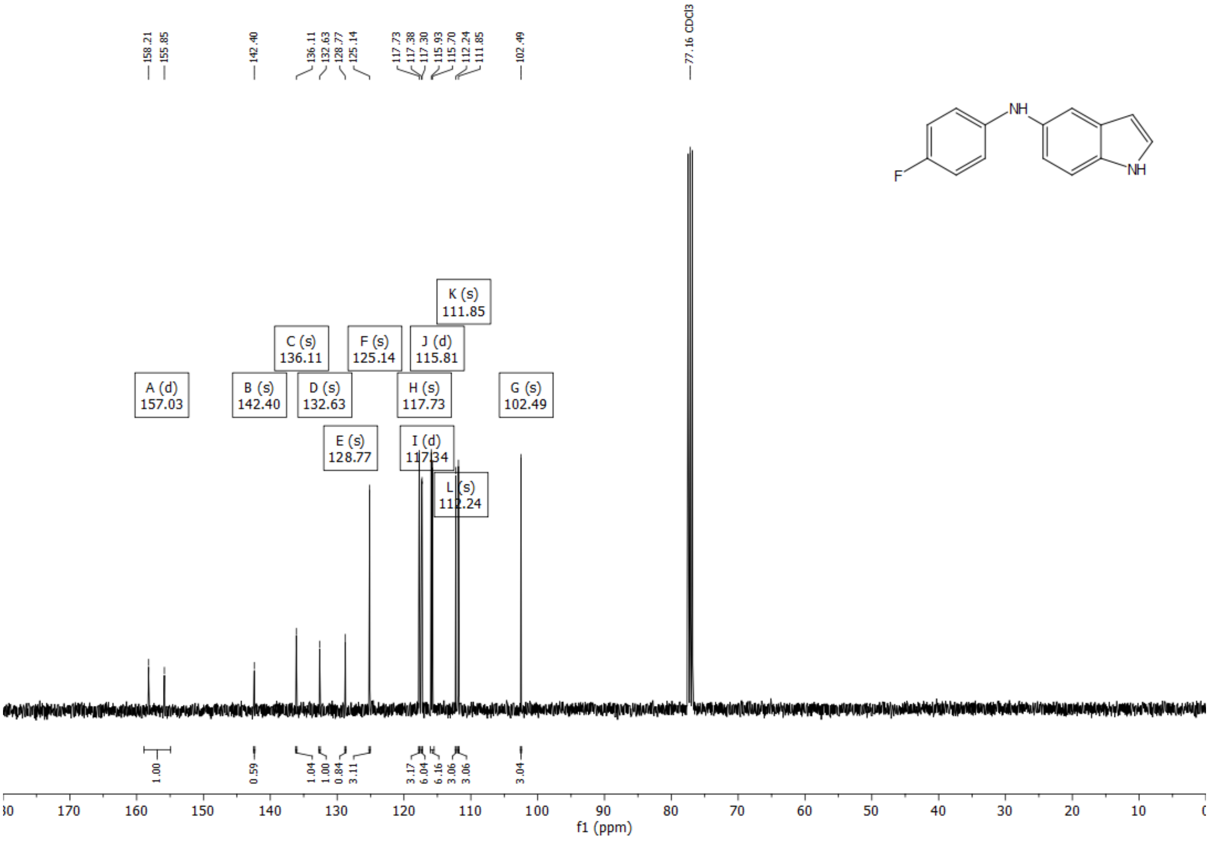

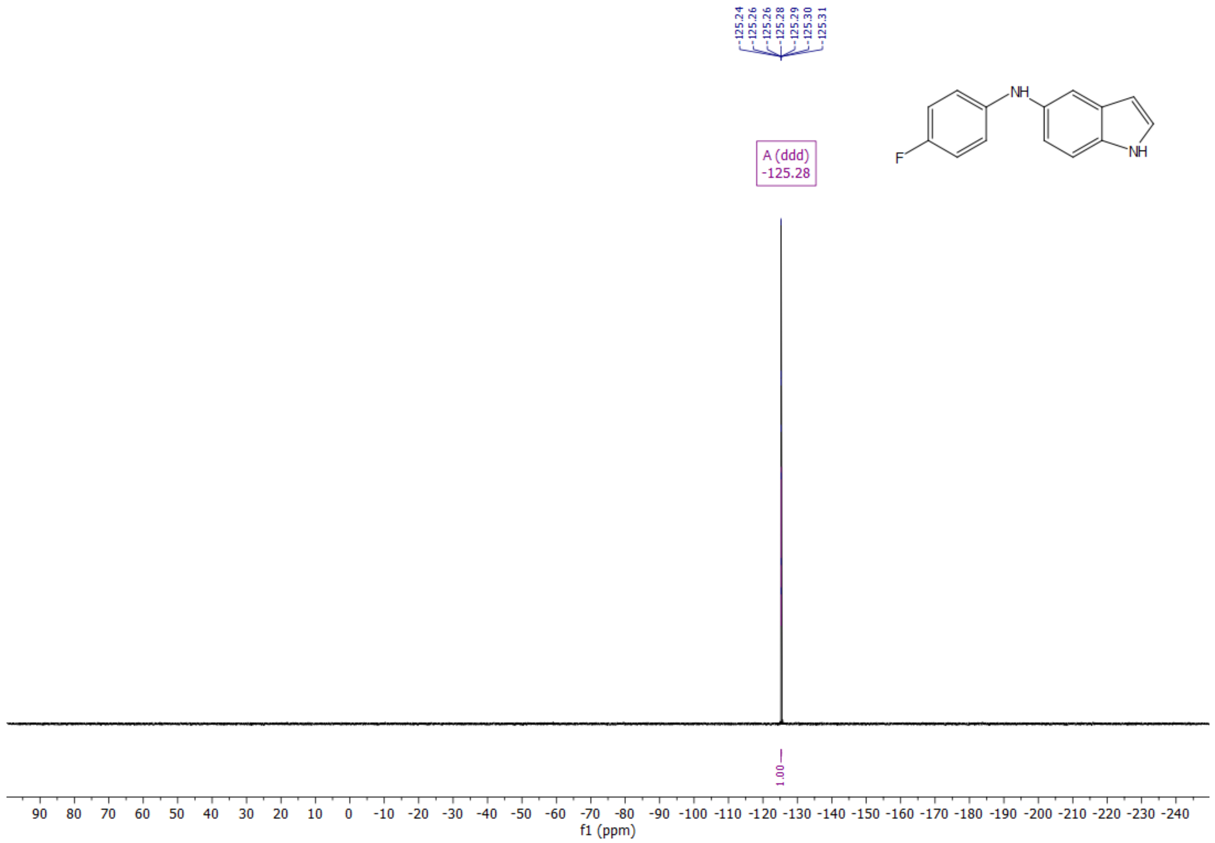

-125.24
-125.26
-125.26
-125.28
-125.29
-125.30
-125.31
A (ddd)
-125.28
NH
F
NH
1.00
90 80 70 60 50 40 30 20 10 0 -10 -20 -30 -40 -50 -60 -70 -80 -90 -100 -110 -120 -130 -140 -150 -160 -170 -180 -190 -200 -210 -220 -230 -240
f1 (ppm)

NL: 4.15E7
ESI79729 #14-25 RT: 0.16-0.29 AV: 6 NL: 4.15E+007
T: FTMS {1,1} + p ESI Full lock ms [80.00-1600.00]

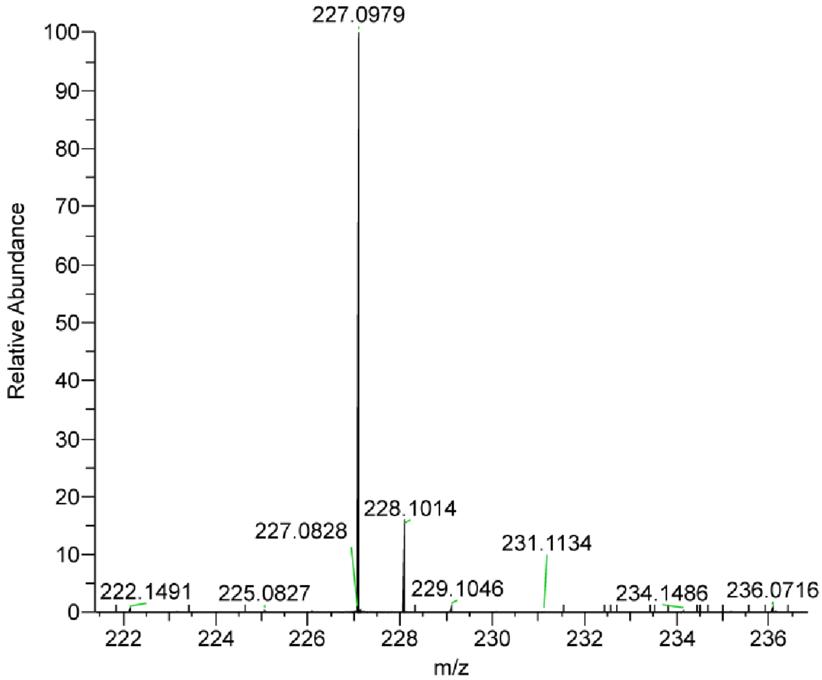


NL: 8.53E5
C14H12N2F Chrg 1 R: 51798 Res. Pwr. @FWHM

**Theoretical Spectrum**

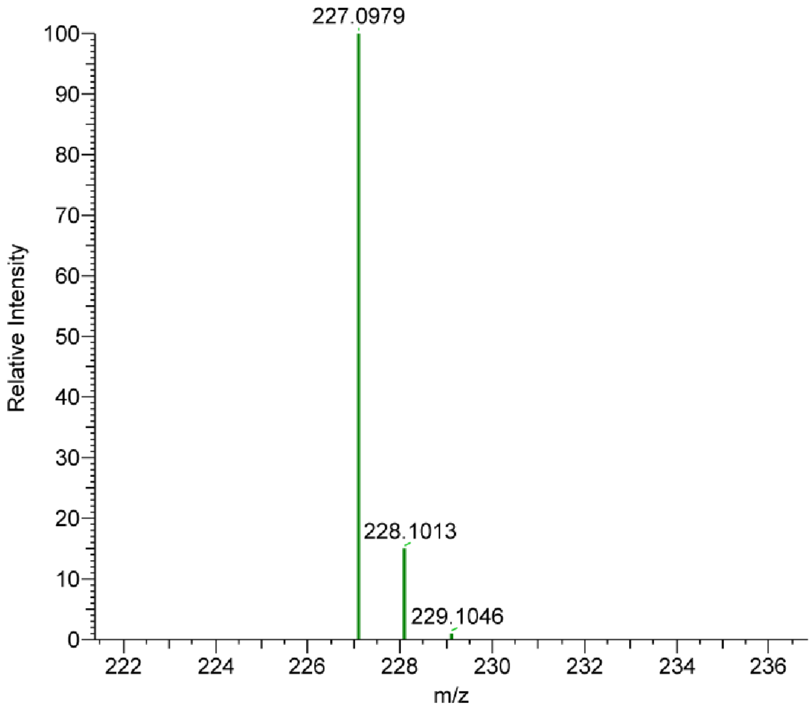


| Peak Mass | Display... | Combin... | RDB | Delta [p... | Theo. m... | Rank | Combin... | # Match... | # Misse... | MS Cov.... | Pattern... | MSMS... |
|---|---|---|---|---|---|---|---|---|---|---|---|---|
| 227.0979 | $C_{14}H_{12}$... | 66.765... | 9.50 | 0.12 | 227.09... | 1 | 91.772... | 3 | 0 | 93.161... | 100 | (Collec... |

**1-Methyl-1*H*-indol-5-amine (11)**

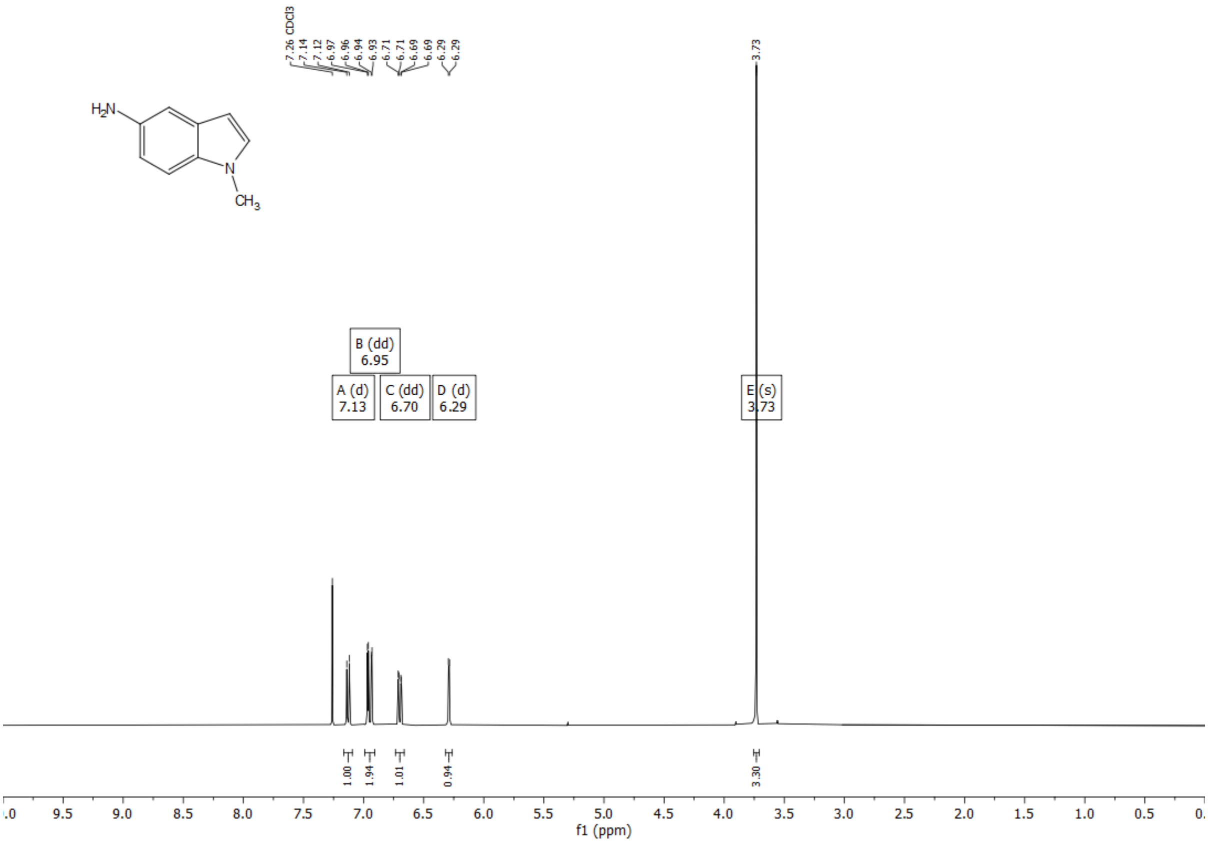


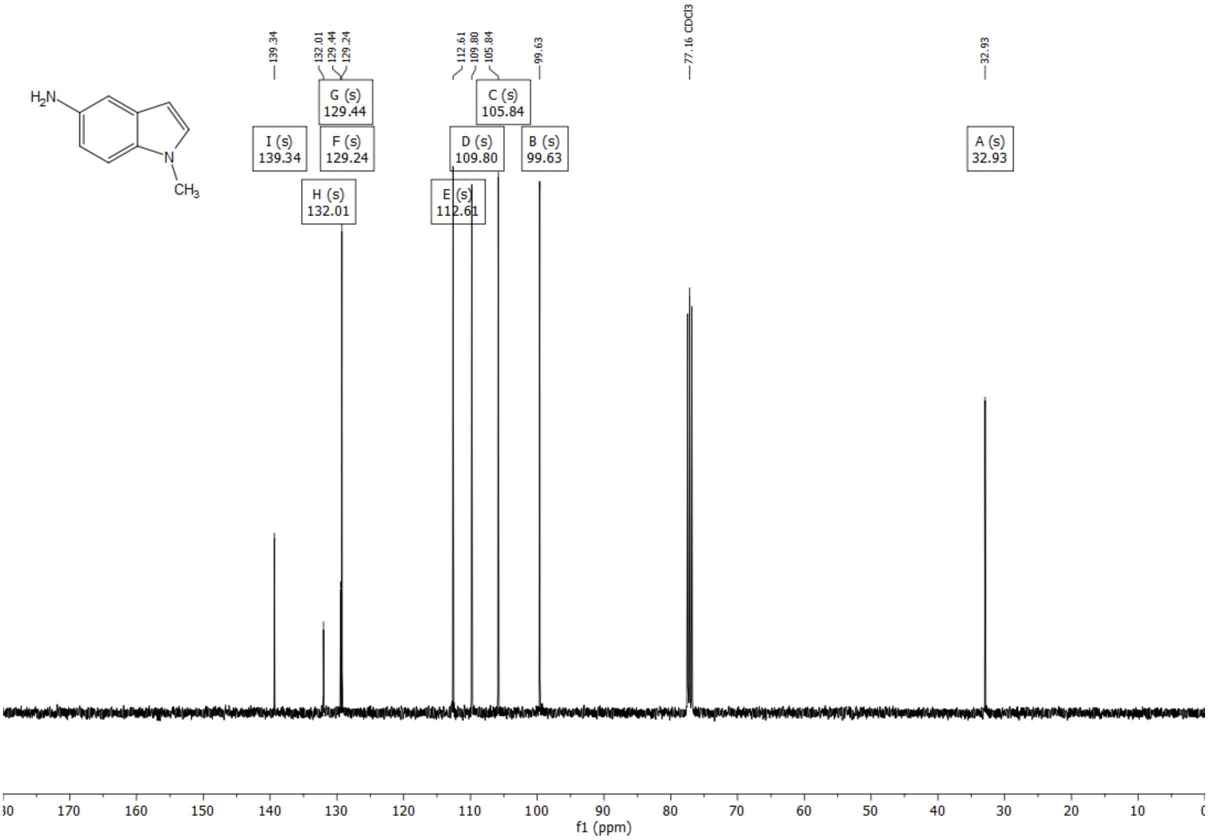

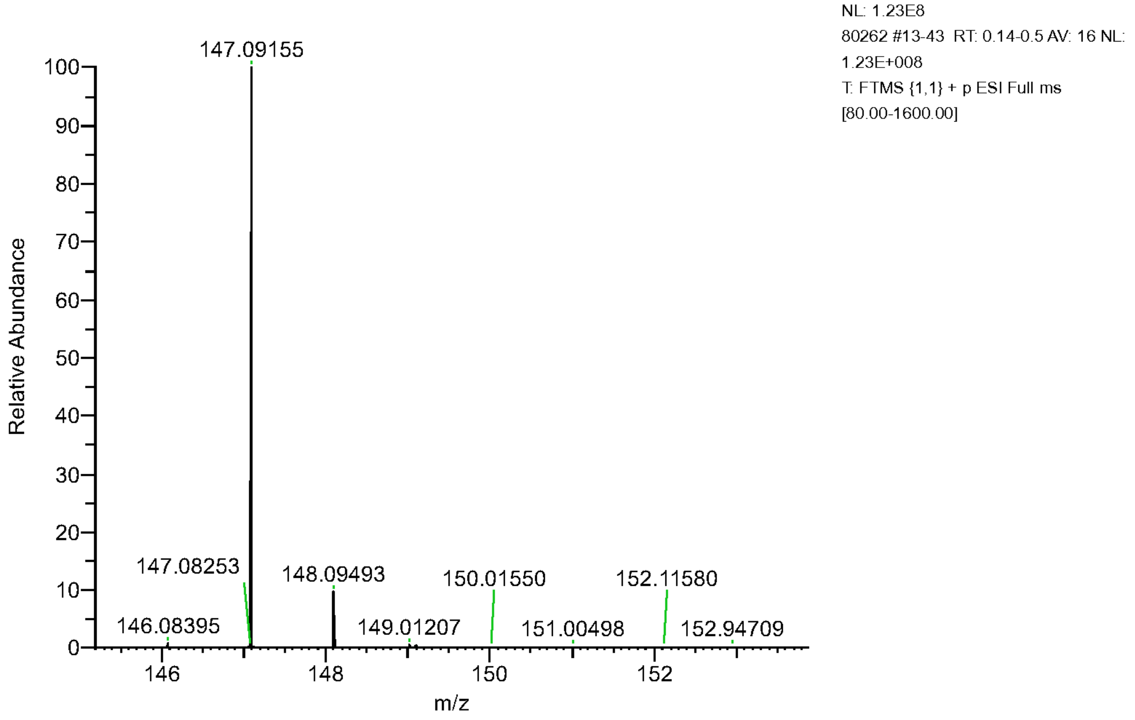


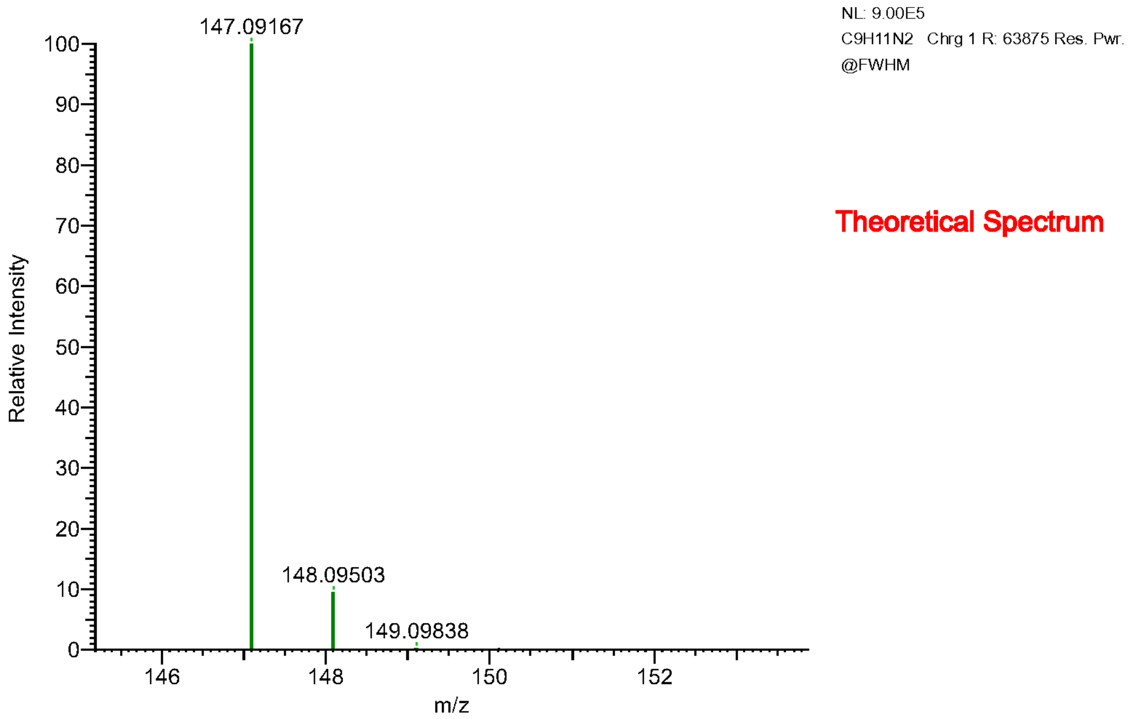


| Peak Mass | Display Formula | Delta [ppm] | Theo. mass | Rank | MSMS Matched Frag... |
|---|---|---|---|---|---|
| 147.09155 | $C_9H_{11}N_2$ | -0.88 | 147.09167 | 1 | (Collection) |
